# Pharmacotherapy alleviates pathological changes in human direct reprogrammed neuronal cell model of myotonic dystrophy type 1

**DOI:** 10.1371/journal.pone.0269683

**Published:** 2022-07-01

**Authors:** Mougina K. Eltahir, Masayuki Nakamori, Satoshi Hattori, Takashi Kimura, Hideki Mochizuki, Seiichi Nagano

**Affiliations:** 1 Department of Neurology, Osaka University Graduate School of Medicine, Suita, Osaka, Japan; 2 Department of Biomedical Statistics, Osaka University Graduate School of Medicine, Suita, Osaka, Japan; 3 Department of Neurology, Hyogo Medical University, Nishinomiya, Japan; University of Louisville, UNITED STATES

## Abstract

Myotonic dystrophy type 1 (DM1) is a trinucleotide repeat disorder affecting multiple organs. However, most of the research is focused on studying and treating its muscular symptoms. On the other hand, despite the significant impact of the neurological symptoms on patients’ quality of life, no drug therapy was studied due to insufficient reproducibility in DM1 brain-specific animal models. To establish DM1 neuronal model, human skin fibroblasts were directly converted into neurons by using lentivirus expressing small hairpin RNA (shRNA) against poly-pyrimidine tract binding protein (PTBP). We found faster degeneration in DM1 human induced neurons (DM1 hiNeurons) compared to control human induced neurons (ctrl hiNeurons), represented by lower viability from 10 days post viral-infection (DPI) and abnormal axonal growth at 15 DPI. Nuclear RNA foci were present in most of DM1 hiNeurons at 10 DPI. Furthermore, DM1 hiNeurons modelled aberrant splicing of *MBNL*1 and 2, *MAPT*, *CSNK1D* and *MPRIP* at 10 DPI. We tested two drugs that were shown to be effective for DM1 in non-neuronal model and found that treatment of DM1 hiNeurons with 100 nM or 200 nM actinomycin D (ACT) for 24 h resulted in more than 50% reduction in the number of RNA foci per nucleus in a dose dependent manner, with 16.5% reduction in the number of nuclei containing RNA foci at 200 nM and treatment with erythromycin at 35 μM or 65 μM for 48 h rescued mis-splicing of *MBNL*1 exon 5 and *MBNL* 2 exons 5 and 8 up to 17.5%, 10% and 8.5%, respectively. Moreover, erythromycin rescued the aberrant splicing of *MAPT* exon 2, *CSNK1D* exon 9 and *MPRIP* exon 9 to a maximum of 46.4%, 30.7% and 19.9%, respectively. These results prove that our model is a promising tool for detailed pathogenetic examination and novel drug screening for the nervous system.

## Introduction

Myotonic dystrophy type 1 (DM1) is an autosomal dominantly inherited multi-organ disease characterized by myotonia, muscle weakness, cataract, respiratory failure, cardiac abnormalities, endocrine and gastrointestinal dysfunction and neurological disturbance [1]. It is the most common form of adult muscular dystrophy with an estimated prevalence of 1:8000 [2]. DM1 neurological manifestations include cognitive dysfunction, memory impairment, emotional deficiency, apathy, mental disorders and excessive daytime sleepiness [3]. Imaging techniques demonstrated cortical gray matter atrophy in frontal, parietal, temporal and occipital regions with diffused white matter lesions and ventricle dilatation in DM1 brain [4–6].

It is postulated that myopathy and neuropathological changes in DM1 brain is caused by CTG trinucleotide expansion in the 3^’^ untranslated region of dystrophia myotonica protein kinase (*DMPK*) gene located on chromosome 19 and a repeat size of 50 or more is considered pathogenic in DM1. After transcription, the expanded CUG repeats are entrapped in the nucleus leading to the formation of hairpin loops known as nuclear RNA foci which sequester muscle-blind like (MBNL) family proteins responsible for alternative splicing of other genes. Thus, resulting in many mis-splicing events [7–11]. Repeat-associated non-ATG (RAN) translation is another pathological mechanism studied in DM1 where the expanded CUG and CAG repeats in sense and antisense transcripts, respectively, form hairpin loops leading to translation of toxic proteins without an ATG start codon [12].

Several therapeutic approaches have been used for the treatment of DM1 muscular symptoms including: the use of antisense oligonucleotide [13], reduction of CUG transcript level [14], inhibition of MBNL1 sequestration [15–17], cleavage of CUG expanded repeats by CRISPR/CAS9 editing [18] or small molecules [19] and blocking the production of CUG repeats by genome modification [20]. However, drug screening for treatments targeting neurological symptoms is lagging behind due to the lack of a good neuronal model. Although animal models were found to recapitulate many DM1 phenotypes in post-mortem brain tissue, they could not fully recapitulate neuropathological features of DM1. For example, discrepancies in regulating alternative splicing of exons were observed in DM1 animal models. This is mostly attributed to species differences between animals and humans [11, 21, 22]. Also, development of phenotypic brain abnormalities in DM1 animal models takes several months (axonal and dendrites degeneration was observed at 9 months and aberrant splicing at 12 months) [23]. Therefore, it makes drug screening a time-consuming process. Brain autopsies can provide insight into brain pathological changes, yet it is limited by other confounders, such as disease severity or comorbidities by the time of death and it cannot be used for drug screening. Obtaining brain biopsy from living patients is impractical and difficult [24]. In vitro modelling using induced pluripotent stem cells (iPSC) derived neural stem cells provides a valuable tool for studying pathological changes in patients, as well as for treatment development. Nevertheless, direct reprogramming of patients’ fibroblasts into neurons skipping the pluripotent phase provides a good opportunity for modelling neuropathological changes and for drug screening concomitantly with the additional advantage of time and cost saving as compared to iPSC derived neural stem cells. Moreover, in contrast to iPSC derived neural stem cells which revert to their pluripotent state losing their epigenetic signature, induced neurons by direct reprogramming can maintain their epigenetic changes providing an additional advantage for investigating age related diseases like neurodegenerative diseases [25].

Neurons generated by direct reprogramming of human fibroblasts are known as human induced neurons (hiNeurons). Direct reprogramming of patients’ fibroblasts into neurons was successful in modelling some neurological diseases such as ALS [26], Huntington’s disease [27], Alzheimer’s disease [28] and Parkinson’s disease [29]. Trans differentiation of fibroblasts into neurons was achieved by using a combination of transcription factors and NeuroD1 [30], expression of miR-9/9* and miR-124 combined with Ascl1 and Myt1l transcriptional factors and NeuroD2 [31], or by chemical cocktail [28]. Also, induced neurons were generated from mouse fibroblasts by downregulation of poly-pyrimidine tract binding protein (*PTBP*) which occurs during normal brain development. This was achieved by using small hairpin RNA against *PTBP* (sh*PTBP*) which resulted in the collective induction of miR-124 and miR-9, five transcription factors *(*Ascl1, Brn2, Myt1l, Zic1, and Olig2*)* and NeuroD1, all of which synergizes with each other to enhance conversion of fibroblasts into neurons. Induced neurons generated by this method were found to be functional with glutamatergic and GABAergic responses [32]. Since glutamate and GABA neurotransmitters are utilized by cortical projection neurons and cortical interneurons, respectively [33], we thought this method of direct reprogramming will be of importance in modelling DM1 cortical neurons since they are affected in this disease as proved by previous studies [8, 34].

In the present study, we are using direct reprogramming approach for the first time to generate DM1 human neurons from patients’ fibroblasts to model and effectively treat the phenotypic brain abnormalities and aberrant splicing of gene transcripts reported previously in the literature by drug therapy. Our DM1 hiNeurons successfully recapitulated neurodegeneration represented by their lower viability and abnormal axonal outgrowth compared to ctrl hiNeurons. We also found accumulation of nuclear RNA foci (one of the molecular hallmarks of DM1) in DM1 hiNeurons where their treatment with actinomycin D was effective in reducing the number of RNA foci. Additionally, DM1 hiNeurons modelled some aberrant splicing events observed in DM1 brain which were rescued by erythromycin treatment; hence, emphasizing the utility of this model for investigating neuropathological mechanisms and its applicability for drug screening studies.

## Materials and methods

### Lentivirus preparation

As previously described [32], lentiviral shRNA against human *PTBP*1 (Cat# TRCN0000231420, Thermo Scientific) was cloned in the pLKO.1 vector (Cat# 8453; RRID:Addgene_8453). Lentivirus was prepared as previously described [35] with modifications, 1–1.2X10^6^ 293FT cells (Cat# R70007, Thermo Fisher Scientific) were seeded in a 10 cm dish coated by 0.12mg/mL poly-l-lysine hydrobromide (PLL) (Cat# P6282, Sigma) in phosphate buffered saline (PBS) (Cat# T900, Takara) and cultured in DMEM high glucose medium (Cat# D5796, Sigma) containing 9% fetal bovine serum (FBS) (Cat# 10437–028, Gibco) and 1 x penicillin/streptomycin (Cat# 15140122, Gibco). Using Calphos Mammalian Transfection kit (Cat# 631312, Takara), 293FT cells were co-transfected with 2.5 μg VSVG (Cat# 35616; RRID:Addgene_35616, Addgene), 7.5 μg Δ8.9 (Cat# PVT2323, Nova Lifetech Inc.) and 10 μg shRNA against human *PTBP*1 plasmids. After 14–16 h, medium was changed with fresh DMEM containing FBS and 1 x penicillin/streptomycin. Thereafter, lentivirus containing medium was collected at 38, 62 and 86 h after transfection and stored at 4°C until ultracentrifugation time. The viral containing medium was filtered using 0.45 μm Rapid Flow Filter Unit (Nalgene), then centrifuged at a speed of 70,000 x g for 2 h and 20 min at 4°C using ultracentrifuge (Optima L-90K, Beckman Coulter Inc.). The precipitate was resuspended in Neurobasal Medium (Cat# 21103–049, Gibco), transferred to 1.5 mL tube and centrifuged at a speed of 14,000 x g at 4 °C for 5 min to remove any residual cells. Then, the supernatant containing lentivirus was aliquoted and stored at -80 °C.

### Cell culture and direct conversion of fibroblasts into neurons

Healthy and DM1 human skin fibroblasts were purchased from the Coriell Institute for Medical Research (Camden, NJ, USA). Further details are provided in Table 1. Fibroblasts were seeded into 8 wells Lab-Tek II chambered glass slide (Cat# 154534, Nunc), 24-well or 6-well plates (Corning Incorporated Costar) at a density of 2 x 10^4^, 3 x 10^4^ or 1.2 x 10^5^ cells, respectively. Chamber glass was coated with 2.5 μg/mL laminin (Cat# 120–05751, FUJIFILM Wako Pure Chemical Corporation) plus 0.15 mg/mL PLL in PBS for at least 40 min before seeding whereas 24-well and 6-well plates were coated with 0.1mg/mL gelatin (Cat# G1890, Sigma) in distilled water.

**Table 1:** Characteristics of cell lines included in the study.

Cell line	Type	Sex	Race	Age at sampling (years)	Lymphocyte Repeat size	Symptoms
**GM03651**	Control fibroblast	Female	White	25	-	Apparently healthy
**GM23963**	Control fibroblast	Male	White	43	-	Apparently healthy
**GM23971**	Control fibroblast	Male	White	33	-	Apparently healthy
**GM05404**	DM1 fibroblast	Female	White	33	-	mild weakness in hands; gallstones; abdominal pain and frequent diarrhea; weakness and myotonia of grip; mild weakness of foot extension; mild myotonic facies; pseudohypertrophy of the calves; percussion myotonia of the thenar eminences; history of infertility; elevated CK^a^; normal ECG^b^
**GM05163**	DM1 fibroblast	Female	White	36	400 CTG repeats in one allele	Hand cramping present since childhood; difficulty swallowing; myotonic facies; lack of facial expression; myotonia evident with grip and with percussion of the thenar eminence; significant weakness in the hands; marked weakness of neck flexors; weakness in the foot-toe extensors and foot dorsiflexors; difficulty getting pregnant
**GM04033**	DM1 fibroblast	Male	White	48	1000 CTG repeats	Temporal atrophy; clinical myotonia; distal weakness

^a^Creatine kinase

^b^Electrocardiogram.

Direct reprogramming was performed as previously reported [32] with some modifications. Human skin fibroblasts were cultured in DMEM high glucose (Sigma) with 9% FBS and 1 x penicillin/streptomycin (10,000 U/mL, Gibco) and transduced by adding lentivirus for 24 h followed by selection of the converted cells with 5 μg/mL puromycin dihydrochloride (Cat# p9620, Sigma) for 48 h. Then, cells were switched to neuronal medium ([–] L-glutamine Neurobasal Medium (Gibco) supplemented with 1mM sodium pyruvate (Cat# 11360–070, Gibco), 27.5 μM 2-mercaptoethanol (Cat# 21985–023, Gibco), 20 ng/mL FGF2 (Cat# 223-FB/CF, R&D Systems), 2% MACS Neurobrew-21 (Cat# 130-093-566, Miltenyi Biotec)). After 3 days, medium was replaced with fresh neuronal medium. From 8 DPI, the following neurotrophic factors at a concentration of 2.5 or 5 ng/mL were added to neuronal medium: brain-derived neurotrophic factor (BDNF) (Cat# 248-BDB/CF R&D Systems), glial cell line-derived neurotrophic factor (GDNF) (Cat# 212-GD/CF, R&D Systems), ciliary neurotrophic factor (CNTF) (Cat# 257-NT-010/CF, R&D Systems) and neurotrophin-3 (NT-3) (Cat# 267-N3/CF, R&D Systems) and medium was changed every 4 days. Fibroblasts and their induced neurons were incubated in a humidified chamber at 37 °C with 5% CO_2_ supply.

### Ethics statement

This study was carried out according to the approval of the Ethics Committee of Osaka University Graduate School of Medicine (approval No.19172-2; title: Research on establishment of fibroblasts and iN cells from patients with neurological disorders and pathological analysis using them). Human fibroblasts were obtained from the Coriell Institute for Medical Research. The written consent forms are maintained by the Coriell Institute for Medical Research and the authors had no direct contact with the donors.

### Immunocytochemistry

As previously described [32], cells were washed twice with PBS, fixed by adding 4% paraformaldehyde (Cat# 09154–85, Nacalai Tesque, Inc.) for 15 min and then permeabilized by adding 0.1% Triton X-100 (Cat# 9002-93-1, Sigma) in PBS for 15 min at room temperature. Then, cells were washed 3 times by PBS and blocked by adding 3% bovine serum albumin (BSA) (Cat# 2320, Sigma) for 30 min at room temperature. After that, primary antibodies diluted with 3% BSA buffer were added and cells were incubated overnight at 4 °C. Primary antibodies used were mouse anti-TUJ1 (Cat# 801202, RRID:AB_10063408, BioLegend, 1:1000), rabbit anti-TUJ1 (Cat# 802001, RRID:AB_2564645, BioLegend, 1:1000), rabbit anti-MAP2 (Cat# 4542, RRID:AB_10693782, Cell Signaling Technology, 1:200), rabbit anti-GABA (Cat# PA5-32241, RRID:AB_2549714, Invitrogen, 1:1000), mouse anti-glutamate (Cat# G9282, RRID:AB_259989, Sigma-Aldrich, 1:1000), mouse anti-NeuN (Cat# MAB377, RRID:AB_2298772, Millipore, 1:100), rabbit anti-synapsin 1 (Cat# 574778-50UL, RRID:AB_565174, Millipore, 1:500) and mouse anti-neurofilament marker (SMI-312, Cat# 837904, RRID:AB_2566782, BioLegend, 1:250). Next day, cells were washed 3 times by PBS and 3% BSA blocking buffer was used to dilute donkey anti-mouse or anti-rabbit IgG secondary antibodies conjugated with Alexa488 (Cat# A-21202, RRID:AB_141607, Invitrogen, 1:1000; Cat# ab150073, RRID:AB_2636877, Abcam, 1:1000, respectively) or Alexa594 (Cat# A21203, RRID:AB_141633, Invitrogen, 1:1000; Cat# A21207, RRID:AB_141637, Invitrogen, 1:1000, respectively). Cells were incubated with secondary antibodies for 1 h at room temperature in the dark followed by 4 times washing by PBS. Nuclei were stained using DAPI. Images were captured by fluorescence microscope (BZ-X710 all in one fluorescence microscope, KEYENCE).

### Viability study

To compare viability differences over time between ctrl and DM1 hiNeurons, live cell imaging was performed using the bright field/phase of KEYENCE BZ-X710 all in one fluorescence microscope with 10x magnification at the following intervals: 8, 10, 12, 16, 22 and 28 DPI. Each 10 images captured from the top to the end of the culture well represent 1 collection for a total of 4 collections from right to left. For each time point, a total of 10 images per sample replicate from 4 different collections (30 images per sample from three replicates) were included in the analysis to insure good representation of neuronal viability in the whole well. Neurons were considered viable, and thus included in the analysis when they were adherent and exhibited neuronal morphology of expanded cell bodies and projecting neurites regardless of their neurites’ lengths whereas cells clumping together and floating in the medium were considered dead and so excluded from the analysis. Viable cell counting was performed manually using ImageJ (RRID:SCR_003070, The National Institute of Health).

### Axonal degeneration analysis

Ctrl and DM1 human fibroblasts were cultured in 48 wells and converted into hiNeurons by direct reprogramming. At 15 DPI, ctrl and DM1 hiNeurons were co-immunostained with TUJ1 and an axon specific marker (SMI-312) as previously described. Images were captured by KEYENCE BZ-X710 all in one fluorescence microscope at 10x magnification and axonal length measurement was performed using Simple Neurite Tracer plugin provided by ImageJ (The National Institute of Health). Measurements were taken from the end of neuronal soma to the end of axonal shaft. The longest, medium and shortest axons from each photo were included in the analysis. A total of 36 images for each sample (from three replicates) were analyzed, each including measurements of 3 axons by the defined criteria (total 108 axons/sample).

### Cytotoxicity study

To test the tolerability of DM1 and ctrl hiNeurons to actinomycin D (Cat# A9415, Sigma) and erythromycin lactobionate (CAS Number# 3847-29-8), fibroblasts were seeded into 96-well plate at a density of 17.5 x 10^3^ cells and converted into neurons by direct reprogramming. At 8 DPI, concentrations ranging from 25–300 μM of erythromycin were incubated with hiNeurons for 48 h and tolerability of cells was assessed visually using Eclipse Ts2 microscope (Nikon) at 4x magnification. Poor tolerability was defined as increased cellular clumping and floating in the culture medium. The same procedure was applied for ACT except that neurons were incubated at 9 DPI with concentrations ranging from 5–100 nM for 24 h.

After reconstitution of erythromycin and ACT, the required drug concentrations were achieved by diluting the drugs in neuronal medium. For comparison, the same amount of neuronal medium without drug was added as placebo.

### Fluorescence in situ hybridization/ Immunocytochemistry

Visualization of RNA foci was done as previously described [36, 37]. DM1 and ctrl hiNeurons were maintained until 10 DPI in 8 wells Lab-Tek II chambered glass slide and washed once with PBS for 1 min, followed by fixation with 3% paraformaldehyde in PBS for 15 min at room temperature. Then, cells were washed with PBS twice for 5 min each and permeabilized by adding 0.5% Triton X-100 (Cat# 35501–15, Nacalai Tesque) in PBS for 5 min. A pre-hybridization solution consisting of 30% formamide (Cat# 16229–95, Nacalai Tesque) with 2x SCC (Cat# 15557–044, Invitrogen) in distilled water was added on cells for 10 min. The following procedures were carried out while protecting from light. Cells were hybridized by adding 1 ng/μL of 5’ Texas red 2-O-methyl-CAG RNA probe (CAGCAGCAGCAGCAGCAGCA) (IDT) in hybridization buffer (33% formamide, 2x SCC, 2 mM vanadyl complex (Cat# S1402S, New England Biolabs), 0.02% BSA (Cat# 2320, Takara), 70 μg/mL yeast tRNA (Cat# AM7119, Thermo Fisher Scientific) in distilled water) and placed in humidified chamber at 37 °C for 2 h. At the same time, the pre-hybridization solution was placed in hybridization chamber for 2 h at 42°C after which is called post-hybridization solution. Then, cells were washed by adding post-hybridization solution and placed in hybridization chamber for 30 min at 42 °C followed by 1 time wash with 1x SCC for 30 min and additional wash with PBS for 5 min. Thereafter, immunostaining of neuronal cells with TUJ1 antibody was performed as previously described. After completion of immunostaining, chamber was separated from the glass slide and DAPI was added to visualize nuclei before sealing the glass slide. Images for aggregation of RNA foci in neurons were captured at 20x magnification by KEYENCE BZ-X710 all in one fluorescence microscope and the number of nuclear or cytoplasmic RNA foci was counted manually using Keyence BZ-X Analyzer software (version 1.3.1.1). 108 neurons present in 6 different fields were analyzed for each sample replicate (total is 324 neurons for each sample from 3 replicates).

As for treatment study, ACT at a concentration of 100 or 200 nM was added to DM1 hiNeurons at 9 DPI for 24 h followed by the same procedure.

The following equation was used to calculate percentage of nuclear or cytoplasmic RNA foci reduction relative to placebo (P) treated hiNeurons:

%RNAfocireduction=totalNo.ofRNAfocitotalNo.ofcellsP-totalNo.ofRNAfocitotalNo.ofcellsACTtotalNo.ofRNAfocitotalNo.ofcellsP×100 (1)

### RNA extraction, RT-PCR and alternative splicing analysis

For comparison between ctrl and DM1 hiNeurons alternative splicing pattern, hiNeurons were maintained until 10 DPI, washed once with PBS and RNA was extracted using Micro RNA Extraction kit (Cat# 74004, Qiagen) with DNase1 treatment following manufacturer protocol. About 73 ng of RNA from each sample was reverse transcribed simultaneously into cDNA using random hexamers and Superscript IV First Strand Synthesis System (Cat# 18091050, Invitrogen) according to manufacturer’s recommendations.

Desired transcripts were amplified by PCR using 0.3 μM primers flanking exons of interest (See S1 Table) and AmpliTaq Gold 360 Master Mix (Cat# 4398881, Applied Biosystems). The amount of cDNA template constituted 7.5% of the total amplification reaction volume. The following PCR conditions were applied: activation of AmpliTaq Gold 360 Master Mix for 10 min at 95°C, followed by 31, 33 or 35 cycles (as indicated in S1 Table) of denaturation for 30 seconds at 95°C, annealing for 30 seconds at the temperature specified in S1 Table for each transcript and extension for 1min at 72°C and 1 cycle of a final extension for 7 min at 72°C.

Amplified PCR products were analyzed by MultiNA automated microchip electrophoresis system (MCE-202 MultiNA, Shimadzu Manufacturing Co., Ltd.) using DNA-500 kit (Cat# P/N 292-27910-91, Shimadzu). The results were viewed as electropherogram peaks and area under the peak was quantified automatically by MultiNA viewer software.

To calculate percentage exon inclusion (% exon inclusion), the area under the peak (AUC) of exon inclusion isoform was divided by the total area of the peaks.


$$\%\;exon\;inclusion=\frac{AUC\;of\;exon\;inclusion\;}{\left(AUC\;of\;exon\;inclusion\;+\;AUC\;of\;exon\;exclusion\right)}\;\times 100$$ (2)

### Rescuing of mis-splicing study

At 8 DPI, ctrl and DM1 hiNeurons were incubated with 35 or 65 μM erythromycin or placebo for 48 h simultaneously and for each sample about 100 ng of the extracted RNA by the previously explained procedure was simultaneously reverse transcribed into cDNA. After calculating % exon inclusion, the change percentage (% change) relative to placebo (P) treated hiNeurons was calculated as follows:

$$\%\;change=\frac{\;\left(\%\;exon\;inclusion\;P\;treated–\%\;exon\;inclusion\;erythromycin\;treated\right)}{\%\;exon\;inclusion\;P\;treated\;}\;\times 100$$ (3)

While rescue percentage (% rescue) was calculated as previously described [17].


$$\%\;rescue=\frac{\left(\%\;exon\;inclusion\;DM1\;P\;treated–\%\;exon\;inclusion\;DM1\;erythromycin\;treated\right)}{\left(\%\;exon\;inclusion\;DM1\;P\;treated\;-\;\%\;exon\;inclusion\;ctrl\;P\;treated\right)}\;\times 100$$ (4)

### Statistical analysis

Three DM1 and three apparently healthy biological replicates (samples) were used to compare various measures between DM1 and control groups. For each sample, multiple cells, nuclei or axons were counted/measured (as indicated in each figure legend) and then averaged to obtain a single value. Two-tailed unpaired t-test was used to compare between the means of DM1 and control groups. For treatment studies (reduction of RNA foci and rescuing of mis-splicing), repeated measures one-way ANOVA test without multiplicity adjustments was used to compare between the means of three groups: placebo, low or high concentration of the tested drugs. Parametric tests were used based on the assumption of normality as it is not applicable to use normality tests to assess this assumption when sample size is small.

To confirm if results of the aberrant splicing treatment experiments were robust, we applied three approaches: 1) by using repeated measures ANOVA to compare between exon inclusion percentage values (raw data), 2) by using repeated measures ANOVA to compare between the log values of exon inclusion percentage or 3) by using repeated measures ANOVA to compare between the percentage of change values instead of exon inclusion percentage values. The three approaches proved statistical significance; thus, p values of the first approach were reported because it depends on raw data and resembled the results of at least one of the other selected approaches.

Experiments were performed in triplicate. Analysis was performed by GraphPad Prism9 (RRID: SCR_002798, GraphPad Software Inc) and the results were considered statistically significant when p<0.05.

## Results

### Direct reprogramming of healthy and DM1 human skin fibroblasts into neurons

Three apparently healthy human skin fibroblasts (GM03651, labeled ctrl 651; GM23963, labeled ctrl 963; GM23971, labeled ctrl 971) and three DM1 human skin fibroblasts (GM05404, labeled DM1 404; GM05163, labeled DM1 163; GM04033, labeled DM1 33) were purchased from Coriell Institute for Medical Research (More information about each cell line is available at Table 1).

**Fig 1:**
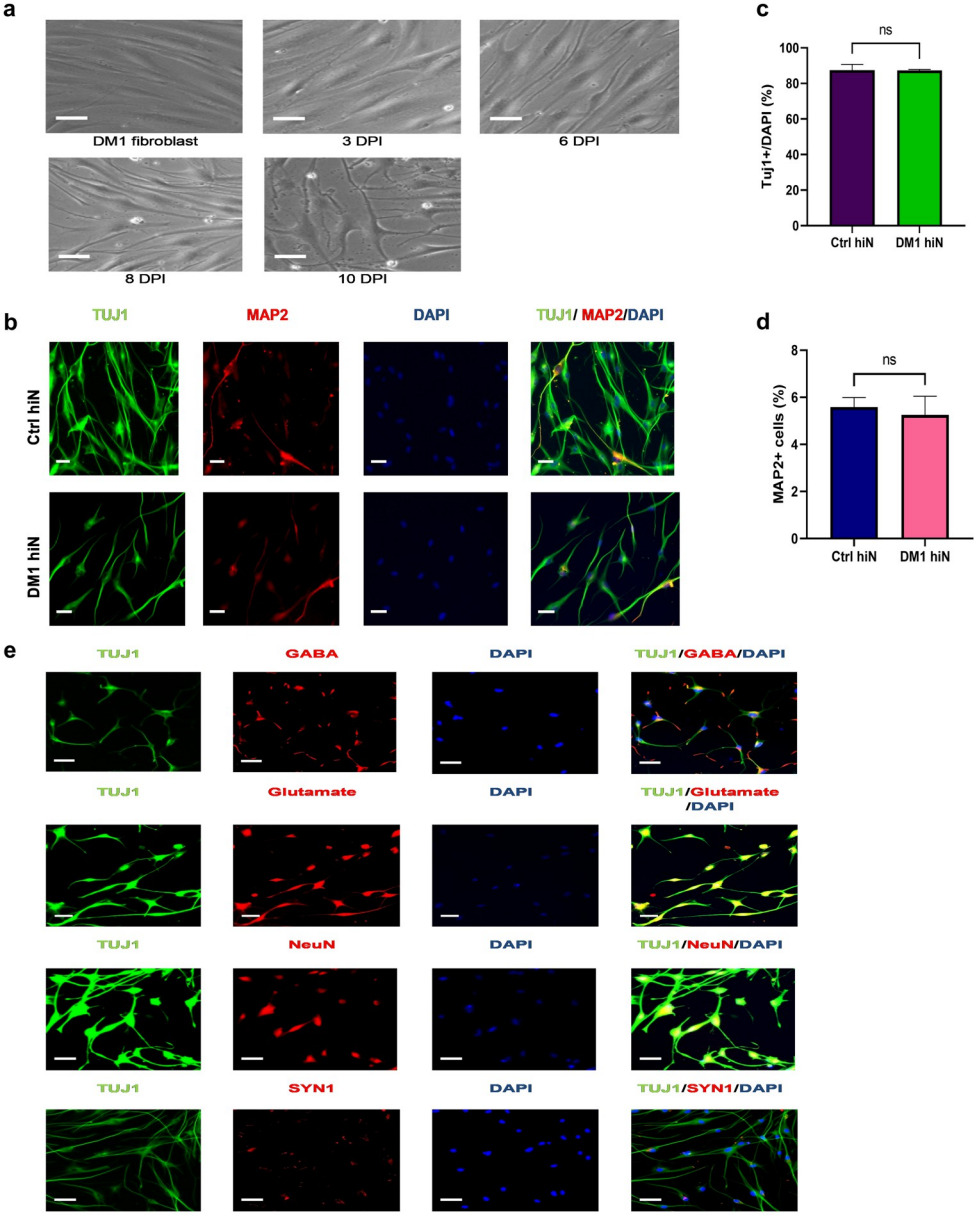
Direct reprogramming of DM1 patients’ skin fibroblasts into neuronal like cells. (a) Differentiation process of DM1 skin fibroblasts into neuronal like cells. The top left image shows DM1 skin fibroblasts before adding lentivirus co-expressing shRNA against *PTBP* and puromycin resistant gene. The top middle image shows lentivirus transduced cells successfully passing the selection process by adding 5 μg/mL puromycin to the culture medium for 2 days. The top right image shows expansion of cell bodies at 6 DPI. The bottom left image shows cells with neuronal like morphology of expanded cell bodies and distinct neurites at 8 DPI. The bottom right image shows bipolar and multipolar neurons with extended neurites at 10 DPI. Scale bar, 50 μm. (b) Co-immunostaining of control (top panel) and DM1 (bottom panel) hiNeurons with a neuron specific β-III tubulin antibody (TUJ1) (green) and MAP2 (red) at 10 DPI. DAPI was used to stain nuclei (blue). On the right: merged images of immunostaining with TUJ1, MAP2 and DAPI. Scale bar, 50 μm. (c) Graph shows the percentage of positive TUJ1-stained cells (TUJ1^+^) at 10 DPI, quantified by dividing the number of TUJ1^+^ cells by the total number of nuclei stained by DAPI. Data are shown as mean ± SEM; n = 3 for each group, a total of 90 images were analyzed per group (11,510 nuclei were analyzed per ctrl group and 14,668 nuclei per DM1 group). ns, not significant compared to control group by unpaired t-test. (d) Graph displays the percentage of MAP2 positive cells at 10 DPI, quantified by dividing the number of MAP2^+^ cells by the total number of TUJ1^+^ cells. Data are shown as mean ± SEM; n = 3 for each group, a total of 90 images were analyzed per group (9,941 TUJ1^+^ cells were analyzed per ctrl group and 16,286 TUJ1^+^ cells per DM1 group). ns, not significant compared to control group by unpaired t-test. (e) Additional staining for neural markers. Fluorescent images display positive co-staining of DM1 hiNeurons with TUJ1 (green) and anti GABA or glutamate or NeuN (red) antibodies at 15 DPI. The bottom panel shows lack of synapse formation at 10 DPI in DM1 hiNeurons co-stained with TUJ1 (green) and SYN1 (red) antibodies. DAPI was used to stain nuclei (blue). On the right merged images. Scale bar, 50 μm. Ctrl, control; DM1, myotonic dystrophy type 1; hiN, human induced neurons; DPI, days post viral-infection; TUJ1, β-III tubulin antibody; MAP2, microtubule-associated protein 2; SEM, standard error of the mean; GABA, gamma-aminobutyric acid; NeuN, neuronal nuclear antibody; SYN1, synapsin 1 antibody.

To generate hiNeurons, healthy and DM1 fibroblasts were transduced with lentivirus co-expressing sh*PTBP* and puromycin resistant gene [32]. Converted neurons were selected by adding puromycin for 2 days. After that, cells were maintained in neuronal medium and at 6 DPI, fibroblasts started to acquire rounded cell bodies, however neurites were still undefined clearly. Since the viability of neurons was poor by 8 DPI in ctrl and DM1 hiNeurons, we decided to add 2-mercaptoethanl and sodium pyruvate as they were shown to improve maintenance of neurons for a longer term [38–40]. Indeed, the viability of neurons have improved at 8 DPI and cells exhibited neuronal like morphology with defined processes. To optimize their development, neuronal growth factors (BDNF, GDNF, NT3 and CNTF) were added to neuronal medium and by 10 DPI, we noticed neuronal like cells with expanded cell bodies and extended neurites. The majority of neurons were bipolar with some multipolar neurons (Fig 1a). Most of these cells showed positive immunoreactivity with TUJ1 anti-body which recognizes the neuronal specific class III β-tubulin antigen [41], and thus confirming successful conversion of fibroblasts into neurons to a similar extent in both groups (Fig 1b and 1c). About 5% of hiNeurons were positive for the mature neuronal marker microtubule-associated protein 2 (MAP2) [42] (Fig 1b and 1d). We confirmed that hiNeurons were composed of a mixture of inhibitory and excitatory neurons as they displayed markers of GABAergic and glutamatergic neurons from 10 DPI when immunostained with gamma-aminobutyric acid (GABA) and glutamate antibodies, respectively (Fig 1e). At 15 DPI, some hiNeurons expressed neuronal nuclear antigen (NeuN) which is a useful marker for assessment of neuronal maturation [43] (Fig 1e). Nevertheless, no synaptic connections were formed between hiNeurons when investigated at 10 or even 17 DPI by immunostaining with synapsin 1 antibody (SYN1) (Fig 1e).

Ctrl and DM1 hiNeurons are composed of a mixture of inhibitory and excitatory neurons as proved by the positive immunoreactivity for GABA and glutamate from 10 DPI. Thus, indicating that this model was successful in generating cortical neurons, the type of neurons that are mostly affected in DM1 [8, 34]. However, this model, showed a small percentage of mature neurons at 10DPI without any synaptic connections. Collectively, these results show that this model is applicable for phenotypic or biochemical studies, however, more optimization will be needed before considering this model for neurophysiological analysis.

### Reduced viability of DM1 hiNeurons

Neuronal loss in frontal and parietal cortices with intensive loss in occipital cortex was reported in DM1 postmortem brain [34]. To investigate if there are any viability differences between ctrl hiNeurons and DM1 hiNeurons over a period of 4 weeks, the same number of ctrl and DM1 human fibroblasts were cultured in 24 wells and converted into hiNeurons using the same lentivirus stock.

We selected 8 DPI as the baseline count of viable neurons in each group because it is the day at which converted cells acquired neuronal like morphology. Viability was monitored at the following intervals: 10, 12, 16, 22 and 28 DPI. We considered neurons as viable and included them in the analysis when they were adherent and exhibited neuronal morphology of expanded cell bodies and projecting neurites regardless of their neurites’ lengths whereas cells clumping together and floating in the medium were considered dead and so excluded from the analysis.

**Fig 2:**
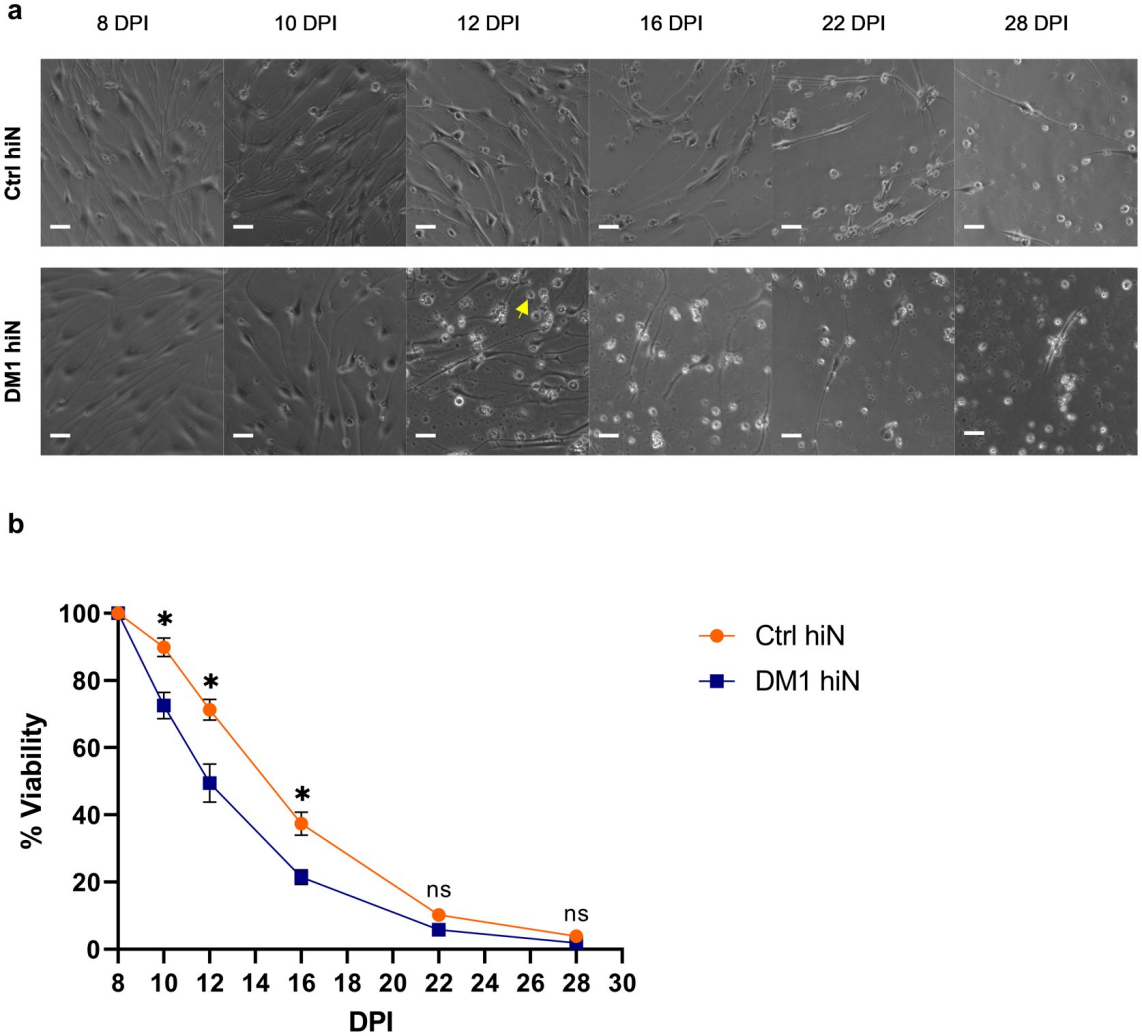
Reduced viability of DM1 hiNeurons. (a) Representative live images demonstrate viability of control and DM1 hiNeurons at the indicated intervals. DM1 hiNeurons (bottom panel) show rapid reduction of cell viability from as early as 10 DPI followed by progressive loss. Arrow indicates dead neurons. Scale bar, 50 μm. (b) Graph shows comparison between the viability of control and DM1 hiNeurons over 4 weeks period relative to their baseline count at 8 DPI (15,773 cells per ctrl group and 24,565cells per DM1 group were counted at baseline). About 50% of DM1 hiNeurons were viable by 12 DPI. Adherent cells were counted manually at the indicated intervals using ImageJ software. Data are presented as mean ± SEM. n = 3 for each group, a total of 90 images were analyzed per group. *P<0.05 and ns, not significant compared to control group by unpaired t-test.

As early as 10 DPI, viability of DM1 hiNeurons dropped to 72.54% compared to 89.87% in ctrl hiNeurons (p = 0.0227). The gap of viability continued to increase at 12 DPI to reach 49.45% against 71.27% in DM1 hiNeurons and ctrl hiNeurons, respectively (p = 0.0273). Remarkably, the viability of DM1 hiNeurons was about half of the ctrl hiNeurons when counted at 16 DPI (21.42% vs. 37.38%, respectively) (p = 0.0176). Viability of DM1 hiNeurons continually dropped to reach 5.80 and 1.88% vs. 10.17 and 3.89% in ctrl hiNeurons at 22 DPI and 28 DPI, respectively (p = 0.1146 and 0.0599, respectively) (Fig 2a and 2b).

The decreased viability observed over time in both groups indicates that most cells are successfully converted into neuronal like cells which were not actively proliferating unlike fibroblasts that are known to proliferate overtime.

### Abnormal axonal outgrowth in DM1 hiNeurons

To verify whether the faster loss of DM1 hiNeurons is associated with any axonal defects, fibroblasts for all cell lines were cultured in 48 wells and the same direct reprogramming protocol was applied. Since we observed a big difference between the viability of ctrl hiNeurons and DM1 hiNeurons between 12 & 16 DPI, we expected that a difference between the two groups may be evident at any time point included in this timeframe. Accordingly, hiNeurons were co-immunostained with SMI-312, a mixture of monoclonal antibodies targeting highly phosphorylated axonal epitopes on neurofilaments M and H [44], and TUJ1 at 15 DPI. The length of axons showing positive immunoreactivity for the axon specific marker (SMI-312) was measured by Simple Neurite Tracer plugin (ImageJ). The longest, medium and shortest axons in each photo were included in the analysis.

**Fig 3:**
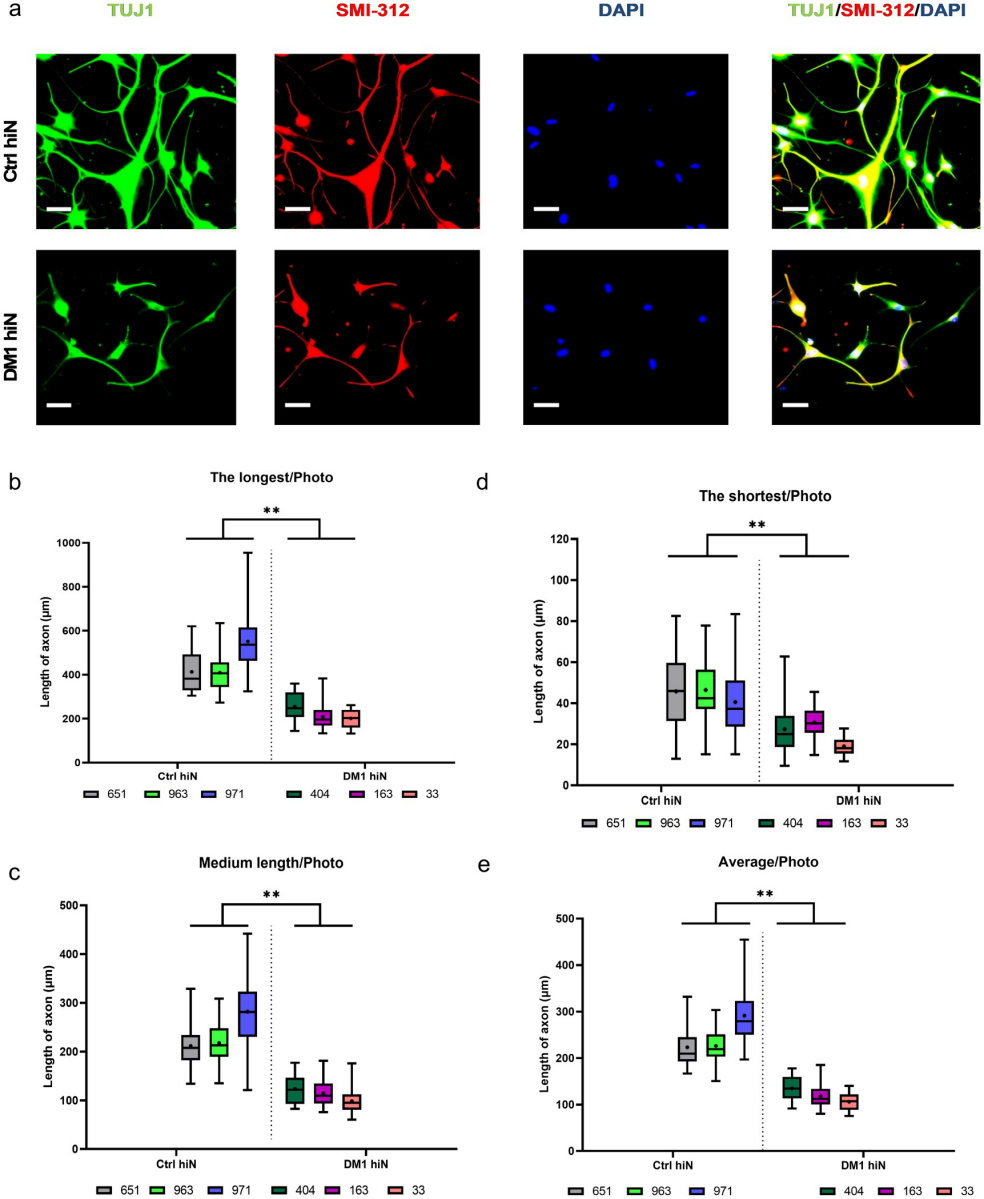
Abnormal axonal outgrowth in DM1 hiNeurons at 15 DPI. (a) Representative fluorescent images display axons of control (top panel) and DM1 (bottom panel) hiNeurons visualized by co-staining with TUJ1 (green) and SMI-312 (red) antibodies. Nuclei stained by DAPI. On the right merged images of TUJ1, SMI-312 and DAPI. Scale bar, 50 μm. (b-e) box and whisker plots show measurements of axonal length of control and DM1 hiNeurons. Results were categorized into four categories: the longest/photo (b), medium length/photo (c), the shortest/photo (d) and average/photo (e). Each sample is shown in different color. Line & (+) sign inside the box represent median and mean of replicates (outcome analyzed), respectively. Whiskers show minimum & maximum values. Note that the average length of axons measured in DM1 hiNeurons group is about half length of axons in control group. n = 3 for each group, a total of 36 (the longest, medium and the shortest/photo analysis) or 108 (average/photo analysis) axons were analyzed per sample. A total of 36 images were analyzed per sample. **P<0.01 compared to control group by unpaired t-test. Average axonal length is the average of the longest, medium and the shortest axons per photo. Medium axon is a single representative of the general population of axons excluding the longest and shortest axons in each photo.

By 15 DPI, the mean of the longest axons measured in DM1 hiNeurons was 221.5 μm compared to 457.9 μm in ctrl hiNeurons (p = 0.0090) while that of the shortest was 25.9 μm in DM1 vs 44.7 μm in ctrl hiNeurons (p = 0.0093). Overall, the axonal length of DM1 hiNeurons was shorter than ctrl hiNeurons with an average length of 119.6 μm in DM1 hiNeurons compared to 247.0 μm in ctrl hiNeurons (p = 0.0058) (Fig 3a–3e).

The faster reduction in the viability of DM1 hiNeurons and their abnormal axonal extension compared to ctrl hiNeurons prove that this model can recapitulate neurodegeneration and brain atrophy observed in DM1 patients [45], post-mortem brain tissue [4–6], and a mouse model [23].

### Accumulation of nuclear RNA foci in DM1 hiNeurons

To confirm the accumulation of expanded CUG repeats as RNA foci in the nuclei of DM1 hiNeurons, fibroblasts of controls and DM1 patients were cultured in chambered glass slide and induced into neurons by the same protocol. At 10 DPI, fluorescence in situ hybridization (FISH) was performed using 5’ Texas red 2-O-methyl- CAG RNA probe followed by immunostaining with TUJ1 and DAPI.

**Fig 4:**
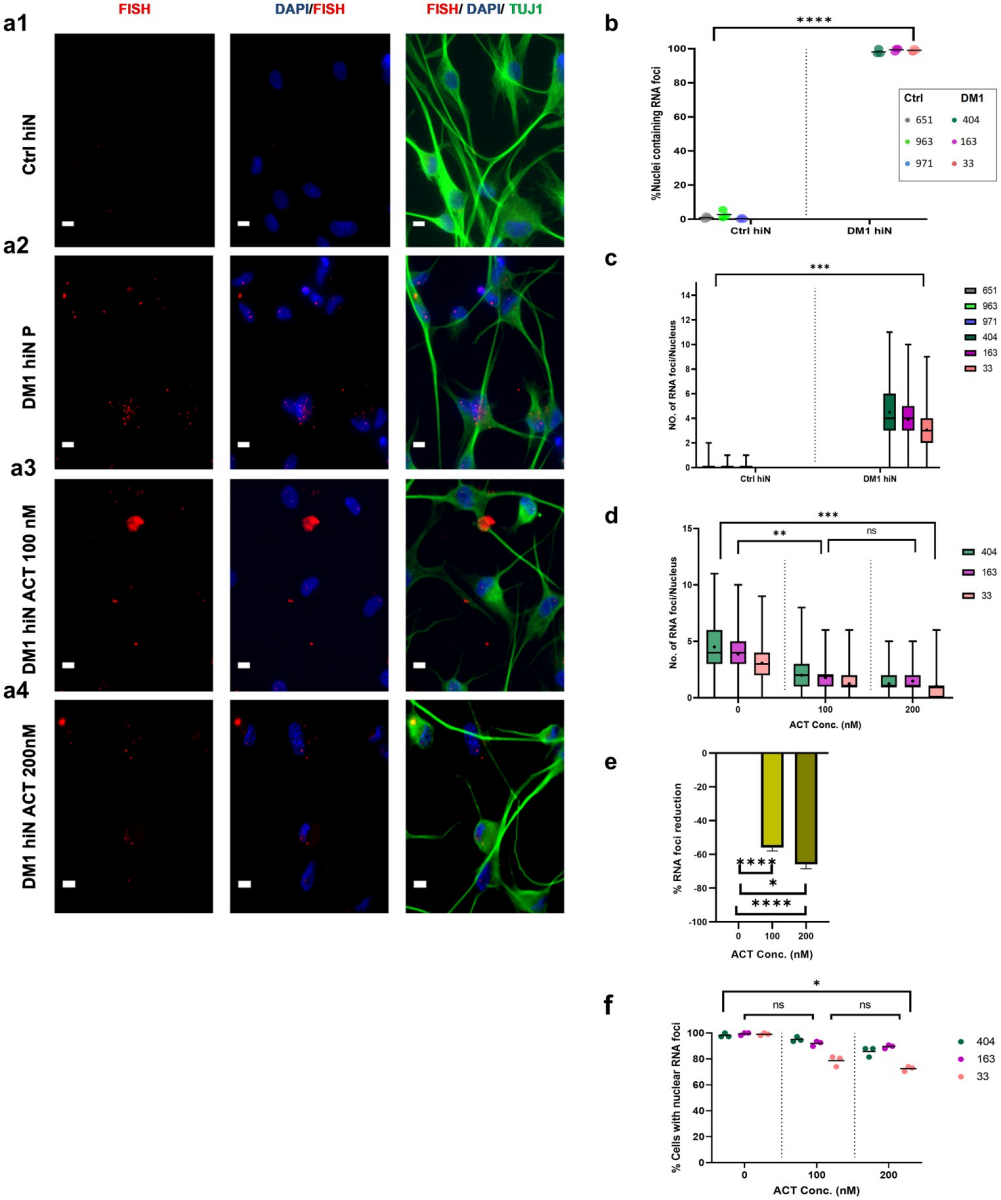
Accumulation of nuclear RNA foci in DM1 hiNeurons at 10 DPI and their reduction by ACT treatment. (a) FISH with 5’ Texas red 2-O-methyl- CAG RNA probe and immunostaining with TUJ1 and DAPI revealed punctate and discrete nuclear RNA foci (red) in DM1 hiNeurons (a2) but not in control hiNeurons (a1). Nuclear RNA foci were abundant in DM1 hiNeurons (a2) but treatment with 100 nM (a3) or 200 nM (a4) ACT reduced them remarkably (left: FISH, middle: merged images of FISH and DAPI staining nuclei (blue), right: merged images of FISH, DAPI and TUJ1 antibody staining neurons (green)). Scale bar, 20 μm. (b) Scatter plot shows percentage of nuclei containing RNA foci in DM1 hiNeurons. Each sample is presented in different color. Each symbol represents the percentage of nuclei containing RNA foci for each sample replicate. Line represents the mean. Note that nuclear RNA foci were present in most of DM1 hiNeurons. (c) Box and whisker plot shows number of RNA foci per nucleus in DM1 hiNeurons. Each sample is presented in different color. Line and (+) sign inside the box represent median and mean of replicates (outcome analyzed), respectively. Whiskers show minimum & maximum values. Counting was performed manually. n = 3 for each group, a total of 324 nuclei were analyzed per sample. ****P<0.0001 and ***P<0.001 compared to control group by unpaired t-test. (d) Box and whisker plot displays the results of nuclear RNA foci count in placebo and ACT treated DM1 hiNeurons. Each sample is presented in different color. Line and (+) sign inside the box represent median and mean of replicates (outcome analyzed), respectively. Whiskers show minimum & maximum values. Note that the maximum number of nuclear RNA foci in placebo treated DM1 hiNeurons is 11 whereas treatment with 100 nM or 200 nM ACT reduced that number to 8 or 6, respectively. (e) Graph shows percentage of nuclear RNA foci reduction in DM1 hiNeurons by ACT treatment. Nuclear RNA foci count was reduced by 56% or 66% in 100 nM or 200 nM ACT treated DM1 hiNeurons, respectively. The reduction percentage is calculated according to Eq (1). (f) Scatter plot shows percentage of nuclei containing RNA foci in placebo and ACT treated DM1 hiNeurons. Each sample is presented in different color. Each symbol represents the percentage of nuclei containing RNA foci for each sample replicate. Line represents the mean. ACT treatment reduced the percentage of nuclei with RNA foci by 10.3% or 16.5% at 100 nM or 200 nM ACT, respectively. Counting was performed manually. n = 3 for each group, a total of 324 nuclei were analyzed per sample. ****P<0.0001, ***P<0.001, **P<0.01, *P<0.05 and ns, not significant compared by repeated measures one-way ANOVA test. P, placebo (same amount of diluent without drug).

Results confirmed the presence of nuclear RNA foci in most of DM1 hiNeurons in contrast to the negligible presence of RNA foci in controls (Fig 4a1, 4a2 and 4b). The average count of RNA foci per nucleus in DM1 hiNeurons was 3.82 whereas the average observed in their counterparts was 0.007 (p = 0.0008) (Fig 4c). Furthermore, RNA foci were also present in the cytoplasm of DM1 hiNeurons (Fig 4a2). However, cytoplasmic RNA foci were fewer than nuclear RNA foci with an average of 0.64 cytoplasmic RNA foci per DM1 hiNeuron (p = 0.0199) (S1a Fig).

Thus, this model was successful in recapitulating accumulation of nuclear RNA foci in DM1 neurons, a neuropathological feature observed in DM1 post-mortem brain tissue [8] and animal [23] and iPSC derived neural stem cells models [20]. Although cytoplasmic RNA foci were not reported in DM1 brain, their presence in this model could be attributed to the existence of cytoplasmic RNA foci in DM1 fibroblasts, the origin from which hiNeurons are derived, as reported previously [46]. Nonetheless, it was found that cytoplasmic RNA foci are not sufficient to provoke DM1 pathological features [46].

### Treatment by actinomycin D reduces nuclear RNA foci in DM1 hiNeurons

To test if nuclear RNA foci in DM1 hiNeurons can be abolished or decreased with drug therapy, we thought to use actinomycin D (ACT) also known as dactinomycin, a drug that was previously reported to reduce the number of RNA foci per nucleus by 50% in DM1 HeLa cell model when added at a concentration of 10 nM for 18 hours [14].

Accordingly, several concentrations up to 100 nM of ACT were initially tested for 24 h to dissolve or reduce nuclear RNA foci in DM1 hiNeurons by 10 DPI. Surprisingly, the previously reported concentration of 10 nM did not show any effect in DM1 hiNeurons but a concentration of 100 nM was effective in reducing RNA foci. To determine if a higher concentration will be more effective, we compared the effect of ACT treatment at 100 nM with 200 nM for 24 h. FISH study at 10 DPI followed by immunostaining revealed that 100 nM of ACT was effective in reducing the number of RNA foci per nucleus by 56% while 66% reduction was achieved by doubling the concentration (p<0.0001 for both concentrations). The mean effect difference between the two concentrations was also significant (p = 0.0256) (Fig 4a3, 4a4, 4d and 4e). Furthermore, treatment with 200 nM ACT reduced the number of nuclei containing RNA foci by 16.5% (p = 0.0199) (Fig 4f). Although some cellular toxicity was observed at 200 nM, it was not severe to exclude its use in the treatment study as increased cellular death is expected with chemo-therapeutic agents like ACT [47] (S2 Fig). Also, treatment of DM1 hiNeurons with 100 nM or 200 nM ACT reduced cytoplasmic RNA foci by 15.75% or 46.85%, respectively. However, the results were statistically insignificant (p = 0.1500, p = 0.0651, respectively) (S1b–S1d Fig).

Our results showed dose dependent effect of ACT on the treatment of DM1 hiNeurons, however, it was difficult to achieve complete dissolution of nuclear RNA foci.

### Dysregulated alternative splicing in DM1 hiNeurons

To explore whether DM1 hiNeurons can model abnormal splicing events previously observed in DM1 post-mortem brain, RNA was extracted from DM1 and ctrl hiNeurons at 10 DPI followed by simultaneous synthesis of cDNA for all samples by reverse transcriptase- polymerase chain reaction (RT-PCR). Then, the desired gene transcripts were amplified using previously published primers flanking exons of interest (See S1 Table). PCR products were analyzed by MultiNA automated microchip electrophoresis system and percentage of exon inclusion was calculated to compare inclusion of alternatively spliced exons between the two groups.

**Fig 5:**
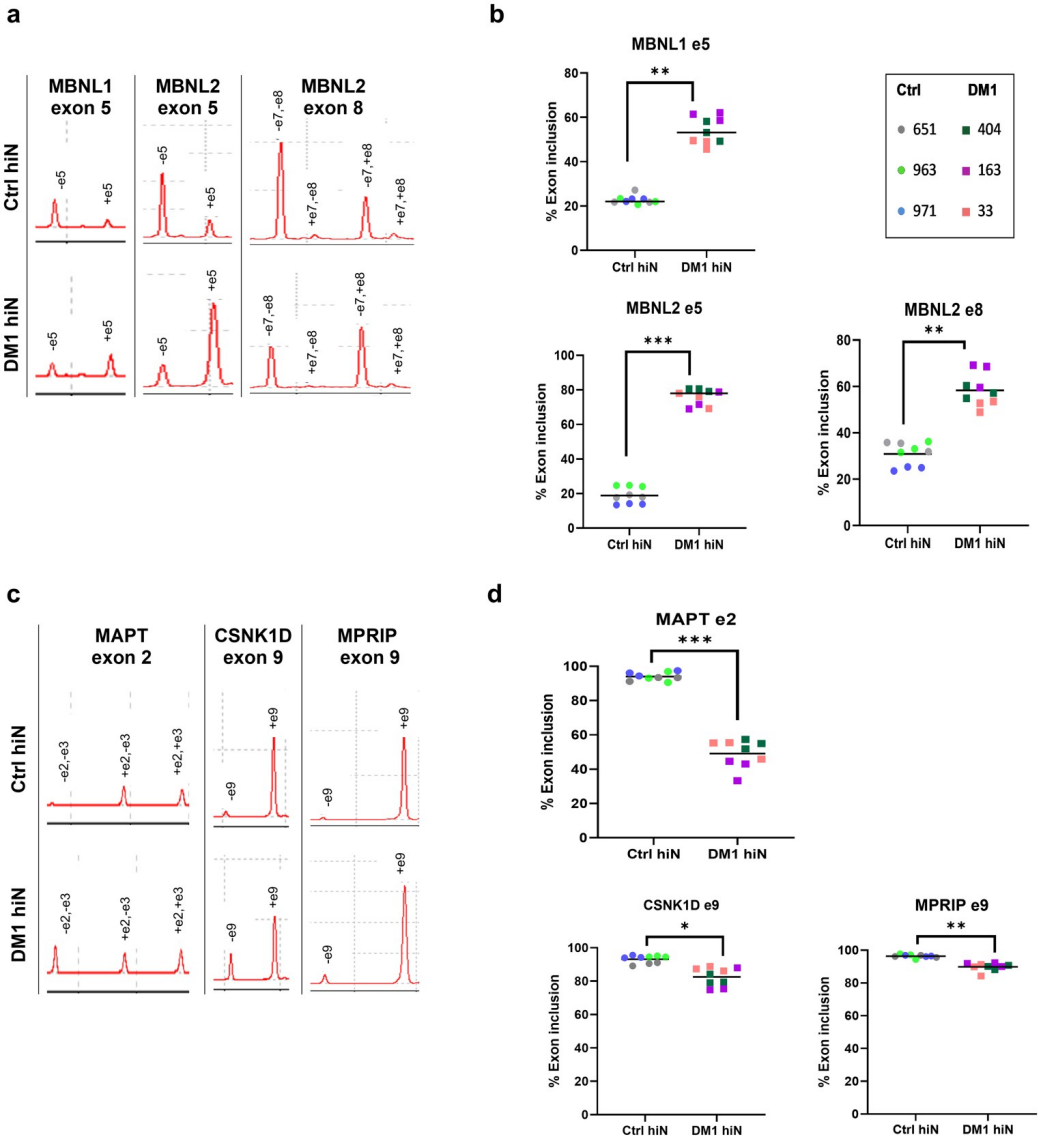
Dysregulated alternative splicing in DM1 hiNeurons at 10 DPI. (a) Electropherograms plot the results of RT-PCR products for *MBNL* 1 and 2 analyzed by MultiNA automated microchip electrophoresis system. Exon inclusion (+) or exclusion (-) of *MBNL*1 e5 (left), *MBNL*2 e5 (middle) and *MBNL*2 e8 (right) in ctrl hiNeurons (top) vs. DM1 hiNeurons (bottom). (b) Scatter plots pertaining to Fig 5a show increased inclusion of *MBNL*1 e5 (top), *MBNL*2 e5 (bottom left) and *MBNL*2 e8 (bottom right) in DM1 hiNeurons. (c) Electropherograms plot the results of RT-PCR products for some gene transcripts involved in neurite outgrowth. Exon inclusion (+) or exclusion (-) of *MAPT* e2 (left), *CSNK1D* e9 (middle) and *MPRIP* e9 (right) are shown. (d) Scatter plots pertaining to Fig 5c show decreased inclusion of *MAPT* e2 (top), *CSNK1D* e9 (bottom left) and *MPRIP* e9 (bottom right) in DM1 hiNeurons. DNA fragment sizes for each transcript are shown in S1 Table. n = 3 for each group. Each sample is presented in different color. Each symbol represents percentage of exon inclusion for one sample replicate. Line represents mean. Area under the peak was used for calculating percentage exon inclusion. *P<0.05, **P<0.01 and ***P<0.001 compared to control group by unpaired t-test. RT-PCR, reverse transcriptase polymerase chain reaction; *MBNL*, muscleblind-like proteins; e, exon*; MAPT*, microtubule associated protein tau; *MPRIP*, myosin phosphatase Rho interacting protein; *CSNK1D*, casein kinase 1 delta.

We found preferential inclusion of exon 5 of *MBNL* 1 and 2 (p = 0.0010 and p = 0.0001, respectively), as well as increased inclusion of exon 8 of *MBNL*2 in DM1 hiNeurons (p = 0.0060) (Fig 5a and 5b). These results are in accordance with the previously published data in DM1 post-mortem brain tissue [10, 48]. Since our model was successful in recapitulating the aberrant splicing of *MBNL*1 and 2 which are known to regulate alternative splicing of other genes, we further investigated alternative splicing of transcripts, specifically those involved in neurite outgrowth as our previous results indicated abnormal axonal extension in DM1 hiNeurons. Accordingly, alternative splicing of microtubule associated protein tau (*MAPT*), myosin phosphatase Rho interacting protein (*MPRIP*) and casein kinase 1 delta (*CSNK1D*) was examined.

Inclusion of *MAPT* exon 2 was significantly reduced in all DM1 hiNeurons compared to controls (p = 0.0006) (Fig 5c and 5d), similar to the results observed in post-mortem studies [8, 49, 50] and DM1 neural stem cells [20]. Likewise, significantly decreased inclusion of *CSNK1D* exon 9 and *MPRIP* exon 9 were found in DM1 hiNeurons relative to ctrl hiNeurons (p = 0.0212 and p = 0.0017, respectively) (Fig 5c and 5d), resembling previous observations in post-mortem DM1 brain tissue [11, 22, 48].

Collectively, these results demonstrate the success of DM1 hiNeurons in modelling deregulated alternative splicing described previously. Moreover, the deregulated alternative splicing of *MBNL*1 and 2 resulted in mis-splicing of their dependent transcripts.

### Treatment of DM1 hiNeurons by erythromycin lactobionate rescues mis-splicing of *MBNL*1, *MBNL*2 and their dependent transcripts

Previous studies have shown that treatment of DM1 fibroblasts and myotubes by erythromycin lactobionate could rescue aberrant splicing of *MBNL* 1 and 2 exon 5 and the splicing of other *MBNL* dependent transcripts in a dose dependent manner [16, 17].

**Fig 6:**
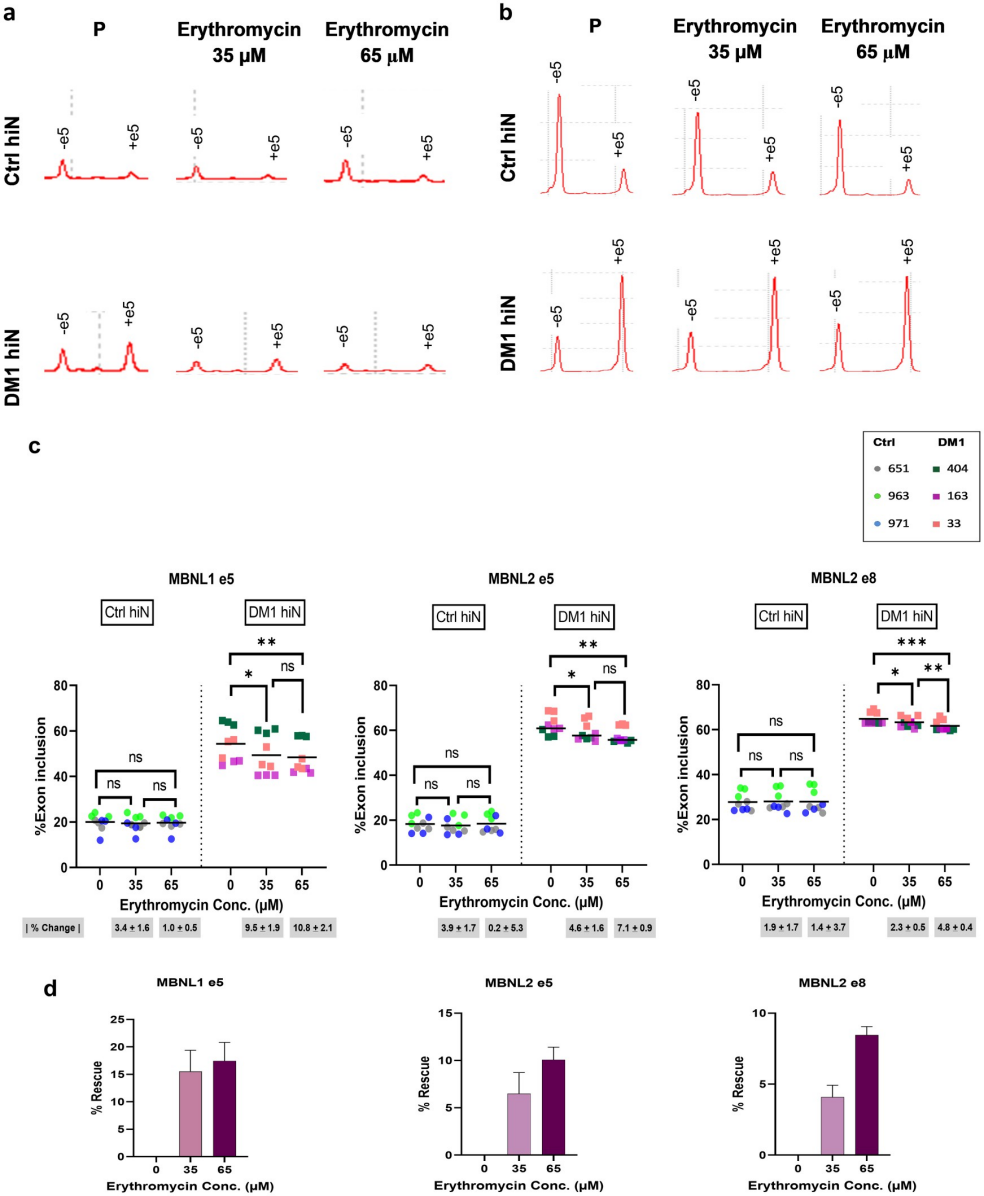
Rescue of *MBNL*1 e5 and *MBNL*2 e5 and e8 mis-splicing by erythromycin lactobionate treatment at 10 DPI. (a-b) Electropherograms plot the results of RT-PCR products for *MBNL* 1 e5 (a) and *MBNL*2 e5 (b) in ctrl (top) and DM1 hiNeurons (bottom). Note the decreased inclusion of *MBNL*1 e5 (+e5) and *MBNL*2 e5 (+e5) in DM1 hiNeurons treated with 35 or 65 μM erythromycin for 48 h relative to placebo treated cells. RT-PCR products were analyzed by MultiNA automated microchip electrophoresis system. (c) Scatter plots show inclusion percentage of *MBNL*1 e5 (left), *MBNL*2 e5 (middle) and *MBNL*2 e8 (right) in control and DM1 hiNeurons treated with placebo or erythromycin. n = 3 for each group. Each sample is presented in different color. Each symbol represents percentage of exon inclusion for one sample replicate. Line represents mean. *P<0.05, **P<0.01, ***P<0.001 and ns, not significant compared by repeated measures one-way ANOVA test. Erythromycin treatment had no adverse effects on control group. Absolute values of mean change percentage |% change| ± SEM relative to placebo treated cells in each group are indicated below the graphs. (d) Bar graphs show rescue percentage of aberrant splicing of *MBNL*1 e5 (left), *MBNL*2 e5 (middle) and *MBNL*2 e8 (right) in DM1 hiNeurons by erythromycin treatment. Data are presented as mean ± SEM. P, placebo (same amount of diluent without drug).

**Fig 7:**
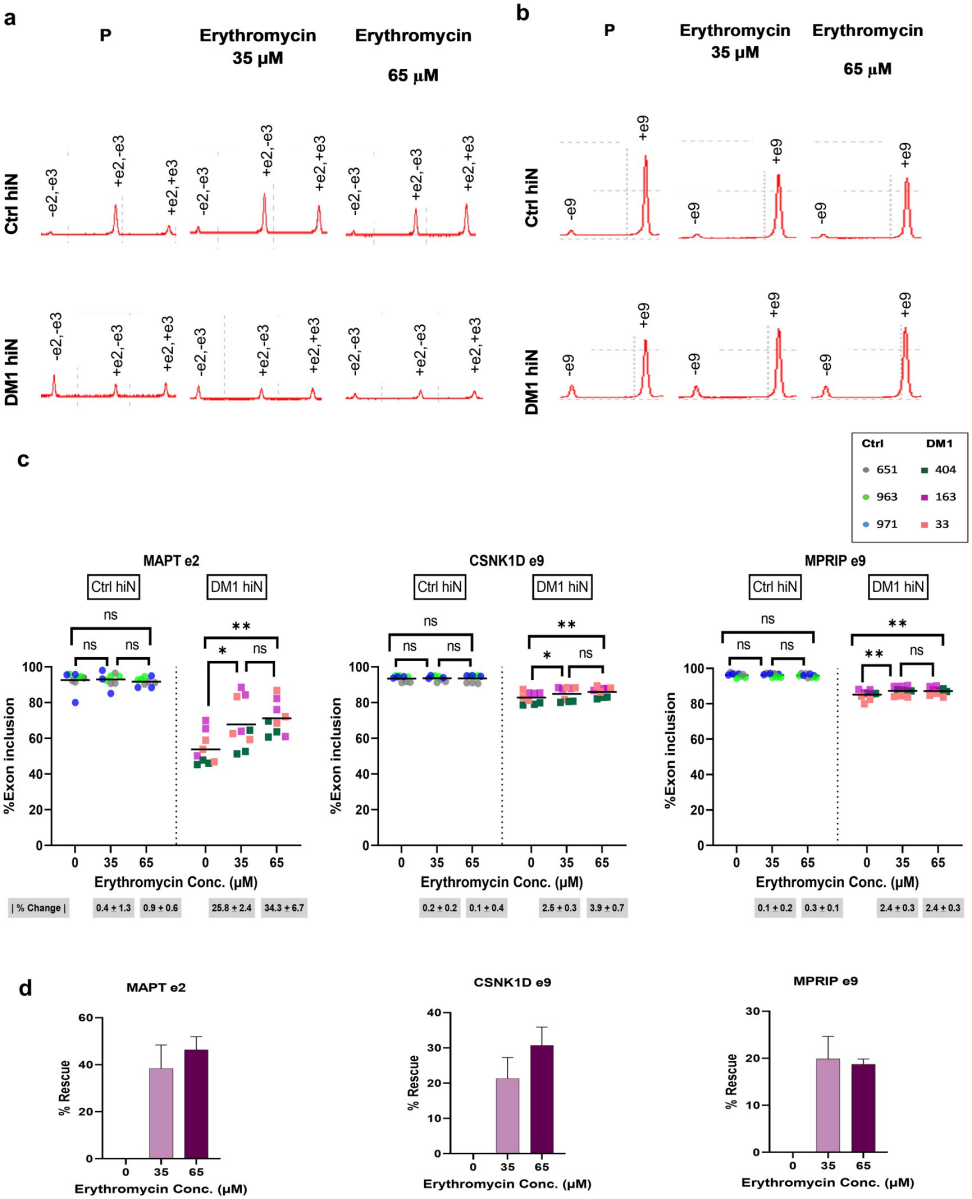
Rescue of *MAPT* e2, MPRIP e9 and CSNK1D e9 mis-splicing by erythromycin lactobionate treatment at 10 DPI. (a-b) Electropherograms plot the results of RT-PCR products for *MAPT* e2 (a) and *CSNK1D* e9 (b) in ctrl (top) and DM1 hiNeurons (bottom). Note the increased inclusion of *MAPT* e2 (+e2, -e3 and +e2, +e3) and *CSNK1D* e9 (+e9) in DM1 hiNeurons treated with 35 or 65 μM erythromycin for 48 h relative to placebo treated DM1 hiNeurons. RT-PCR products were analyzed by MultiNA automated microchip electrophoresis system. (c) Scatter plots show inclusion percentage of *MAPT* e2 (left), *CSNK1D* e9 (middle) and *MPRIP* e9 (right) in control and DM1 hiNeurons treated with placebo or erythromycin. n = 3 for each group. Each sample is presented in different color. Each symbol represents percentage of exon inclusion for one sample replicate. Line represents mean. *P<0.05, **<0.01 and ns, not significant compared by repeated measures one-way ANOVA test. Erythromycin treatment had no adverse effects on control group. Absolute values of mean change percentage |% change| ± SEM relative to placebo treated cells in each group are indicated below the graphs. (d) Bar graphs show rescue percentage of aberrant splicing of *MAPT* e2 (left), *CSNK1D* e9 (middle) and *MPRIP* e9 (right) in DM1 hiNeurons by erythromycin treatment. Note that erythromycin rescued *MAPT* exon 2 mis-splicing by 38.4% or 46.4% at a concentration of 35 or 65 μM, respectively. Data are presented as mean ± SEM. P, placebo (same amount of diluent without drug).

To verify whether similar results can be obtained in DM1 hiNeurons, we tested treatment with several concentrations of erythromycin lactobionate. Our treatment trial showed that treatment with 65 μM erythromycin can improve mis-splicing of *MAPT* exon 2 whereas treatment with a higher concentration of 100 μM was not. Accordingly, DM1 hiNeurons were simultaneously treated with 35 μM or 65 μM erythromycin or placebo for 48 h and RNA was extracted at 10 DPI followed by the same pre-mentioned procedures for alternative splicing study. Interestingly, erythromycin at 35 μM or 65 μM was effective in decreasing inclusion of exon 5 of *MBNL*1 and *MBNL*2 compared to placebo treated DM1 hiNeurons (p = 0.0115, p = 0.0061, respectively for *MBNL*1 and p = 0.0153, p = 0.0032, respectively for *MBNL*2) (Fig 6a–6c). At 65 μM, erythromycin rescued mis-splicing of *MBNL*1 exon 5 by 17.5% and *MBNL*2 exon 5 by 10% (Fig 6d). Similarly, both doses reduced *MBNL*2 exon 8 inclusion (p = 0.0110 at 35 μM, p = 0.0008 at 65 μM). Up to 8.5% normalization of its aberrant splicing was achieved at 65 μM (Fig 6c and 6d). Remarkably, 35 or 65 μM of erythromycin significantly increased *MAPT* exon 2 inclusion (p = 0.0105, p = 0.0044, respectively) (Fig 7a and 7c) with a rescue percentage of 38.4% at 35 μM whereas that of 65 μM was 46.4% (Fig 7d). Furthermore, 35 or 65 μM of erythromycin increased inclusion of *CSNK1D* exon 9 and *MPRIP* exon 9 compared to placebo treated DM1 hiNeurons (p = 0.0258, p = 0.0060, respectively for *CSNK1D* and p = 0.0023, p = 0.0024, respectively for *MPRIP*). A maximum of 30.7% rescue of *CSNK1D* exon 9 and 19.9% rescue of *MPRIP* exon 9 was observed when DM1 hiNeurons were treated with 65 μM or 35 μM erythromycin, respectively (Fig 7c and 7d).

To ensure that the studied doses are not associated with adverse effects, the same concentrations of erythromycin were studied on ctrl hiNeurons and no significant changes were observed on alternative splicing of any of the studied transcripts (Figs 6a–6c and 7a–7c).

These findings confirm that erythromycin treatment was effective in correcting mis-splicing of all studied transcripts. Importantly, no pronounced cellular toxicity was observed at 35 μM but the additional improvement achieved with the higher concentration is accompanied with some additional risk of cytotoxicity (S3 Fig).

## Discussion

Unravelling the underlying neuropathological mechanisms of DM1 and targeting them with therapeutic interventions is essential to improve the quality of life of DM1 patients. Although, DM1 is a multi-organ disease, most of the research is directed toward studying the underlying pathomechanisms of myopathy and based on this knowledge many therapeutic approaches have been employed for the treatment of its muscular symptoms. This could be explained by the availability of animal and in vitro models capable of recapitulating the muscular symptoms. On the other hand, no drug therapy has been studied for the treatment of neurological abnormalities due to insufficient reproducibility in DM1 brain-specific animal models as alternative splicing differences were observed in them. Also, using iPSC derived neural cells for drug screening is limited by its costly and longer procedure. Therefore, the need arises to establish a new in vitro neuronal model.

In this study, direct reprogramming of DM1 patients’ fibroblasts into neurons by the stable knockdown of *PTBP* was used to generate DM1 in vitro neuronal model. DM1 hiNeurons recapitulated features of decreased viability, abnormal axonal outgrowth, nuclear RNA foci accumulation and alternative splicing abnormalities observed in DM1 post-mortem brain tissues and in vitro and animal models. In our study, aberrant splicing of gene transcripts involved in neurite outgrowth preceded axonal outgrowth defects. Furthermore, treatment of DM1 hiNeurons with ACT significantly reduced the number of nuclear RNA foci by more than 50% and treatment with erythromycin rescued mis-splicing of *MBNL*1 exon 5 and *MBNL*2 exons 5 and 8 up to 17.5%, 10% and 8.5%, respectively. Furthermore, erythromycin rescued the aberrant splicing of *MAPT* exon 2, *CSNK1D* exon 9 and *MPRIP* exon 9 to a maximum of 46.4%, 30.7% and 19.9%, respectively. Importantly, the dual studies of viability and measurement of axonal length where rapid decline in viability and abnormal axonal outgrowth were observed in DM1 hiNeurons, indicate that DM1 neurons undergo faster degeneration than ctrl neurons. These results are in accordance with the neurodegeneration phenomenon observed over a decade in DM1 patients [45], and thus explain the finding of cerebral atrophy in DM1 postmortem brain.

DM1 hiNeurons modelled aggregation of nuclear RNA foci and treatment with 100 or 200 nM ACT for 24 h reduced the number of RNA foci per nucleus by 56% or 66%, respectively, with 16.5% reduction in the number of nuclei containing RNA foci by the higher concentration. Similar to the results observed in DM1 HeLa cell model after treatment with 10 nM ACT for 18 h [14]. Both findings indicate the difficulty of complete dissolution of RNA foci by ACT in DM1 in vitro models. In this study, using 10x higher concentrations of ACT to achieve similar results, suggests that neurons are insensitive to low concentrations of ACT. The need for higher doses in non- dividing cells like neurons may be explained by the pharmacodynamic properties of ACT as it is a cell-cycle specific drug where cells in the G_1_-S border phase are most sensitive [51].

DM1 hiNeurons were successful in modelling aberrant splicing of *MBNL*1 exon 5 and *MBNL*2 exons 5 and 8, *MAPT* exon 2, *CSNK1D* exon 9 and *MPRIP* exon 9 as observed in post-mortem DM1 brain but to a lesser extent than what was previously reported for the last three mentioned transcripts [11, 22, 48, 50]. This may be explained by the following reasons: 1) DM1 hiNeurons model current neuropathological changes in DM1 which may be progressively altered at the time of death. 2) The brain is composed of variety of cells. For example, RNA foci were also found in oligodendrocytes of DM1 brain [8] but whether splicing defects occur in these cells is yet unknown. Furthermore, the expression level of tau in oligodendrocytes and astrocytes in DM1 needs further investigation as it was found to play a role in impairment of neuronal network and the spread of pathological tau in a number of neurodegenerative diseases like Alzheimer’s disease, progressive supranuclear palsy and Pick’s disease [52–54]. 3) DM1 hiNeurons are cortical neurons not specified to any brain region and it was shown that neurons obtained from different brain regions from the same patients show some variation in the extent of mis-splicing [55]. Likewise, discrepancies in splicing abnormalities between grey matter and white matter in the same patients have been reported [48]. 4) Individual differences of disease severity. 5) Small sample size in this study. Nevertheless, the differences between ctrl and DM1 hiNeurons remain to be significant; thus, supporting the applicability of this model for drug screening studies.

It was found that preferential inclusion of exon 5 of *MBNL*1 and 2 influence the localization of MBNL proteins in the nucleus and increased inclusion of *MBNL*2 exon 8 is associated with reduced exchange of MBNL proteins between the nucleoplasm and expanded CUG hairpin loops; thus, stabilizing the CUG-MBNL complex [56]. Tau protein is involved in stabilization and assembly of microtubules, transport of vesicles and organelles on microtubules and regulation of cell shape and motility [57–59], and hence play an important role in the maintenance of axonal structure and function. Suppression of tau by antisense oligonucleotide was shown to reduce axonal length in cerebellar neurons compared to sense treated neurons [60]. *CSNK1D* is involved in neurite outgrowth, circadian rhythm generation and Fas*-*mediated apoptosis [61, 62]. The relationship between *CSNK1D* and neurodegeneration has been proven in amyotrophic lateral sclerosis where a pharmacological inhibition of CK1-D (the enzyme encoded by *CSNK1D*) resulted in decreased phosphorylation of TDP-43 *(*TAR DNA-binding protein 43) by this kinase and enhanced preservation of spinal motor neurons [63]. *MPRIP* modulates neurite outgrowth through inactivation of RhoA-Rho kinase (ROCK) actomyosin pathway that signals growth cone collapse and neurite retraction [64]. *MPRIP* mis-splicing can negatively affects its role in promoting neurite outgrowth. For example, *PARK2* knockout human iPSC derived neural cells, a model of Parkinson’s disease, showed that increased Rho signaling was accompanied by decreased levels of phospho-*MPRIP*, impaired neurite outgrowth and increased cell migration whereas pharmacological inhibition of RhoA signaling in this model resulted in improved neurite outgrowth and decreased cell migration [65].

The higher concentration of erythromycin was associated with more rescue of the dysregulated splicing in DM1 hiNeurons than the lower concentration. These data are in concordance with the dose-dependent response observed in DM1 human fibroblasts, human derived myotubes and animal models [16, 17]. Erythromycin was significantly effective in rescuing *MBNL*1 exon 5 and *MBNL*2 exons 5 and 8 with a parallel rescue of their dependent transcripts: *MAPT*, *CSNK1D* and *MPRIP*. In vitro study found that MBNL1 loss induced *MAPT* exon 2 exclusion [10]. To the contrary, single knockout of *MBNL*1 in animal model could not induce mis-splicing of *MAPT* exon 2 but single knockout of *MBNL*2 or double knockout of *MBNL*1 and 2 induced skipping of *MAPT* exon 2 [11, 22]. In our study, correction of *MAPT* exon 2 mis-splicing might be the result of the parallel rescue *of MBNL*1, *MBNL*2 or a cumulative result of the correction of both mis-splicing events. *MBNL*2 is the main regulator of *CSNK1D* alternative splicing as association between depletion of *MBNL*2 and decreased inclusion of *CSNK1D* exon 9 was proved by in vitro and animal studies [11, 21]. Moreover, exon 8 of *MBNL*2 was found to be the strongest regulator of *CSNK1D* alternative splicing in DM1 HeLa model [56]. Hence, correction of CSNK1D exon 9 dysregulated splicing can be attributed to the rescue of *MBNL2* by erythromycin treatment. Aberrant splicing of *MPRIP* exon 9 was also rescued by erythromycin treatment. Previous data showed a lack of significant splicing alteration of *MPRIP* exon 9 in *MBNL*1 knockout mice model [22]. Therefore, its correction is perhaps a ramification of *MBNL*2 mis-splicing rescue by erythromycin.

Actinomycin D is an anti-neoplastic drug currently approved for the treatment of different types of cancer such as Wilms’ tumor and Ewing sarcoma. Furthermore, a clinical study reported significant improvement in time to progression and overall survival of pediatric patients suffering from CNS atypical teratoid rhabdoid tumor when treated by chemotherapy regimen containing ACT [66]. Erythromycin is a macrolide antibiotic currently prescribed for the treatment of a variety of bacterial infections including acne, bronchitis and urethritis. Although erythromycin is not approved for CNS infections, some reports suggest its efficacy in treating CNS infections [67]. Importantly, the observed beneficial effects of ACT and erythromycin in DM1 results from interference with the underlying molecular pathology of the disease where ACT exerts its therapeutic effects by reducing CUG-RNA transcript level [14] whereas erythromycin by direct inhibition of MBNL1 sequestration via competitive binding to the expanded CUG repeats [16].

## Conclusion

Overall, our results provide evidence that DM1 hiNeurons is a promising model for studying and investigating the underlying pathological mechanisms of DM1 brain and it will pave the way for future drug development.

In the future, we hope to employ this model for drug screening and for unravelling the correlation between DM1 splicing dysregulation and phenotypic abnormalities. Also, it will be interesting to explore if this model can reproduce pathological changes of other neurological and psychiatric diseases characterized by involvement of cortical neurons such as multiple sclerosis [68], corticobasal degeneration [69], autism spectrum disorders [70], among others.

## Supporting information

S1 Table List of primers used for RT-PCR. (PDF)

S1 Fig Effect of ACT treatment on cytoplasmic RNA foci in DM1 hiNeurons. (a) Scatter plot shows number of RNA foci per nucleus vs cytoplasm of DM1 hiNeurons. Each symbol represents the mean of RNA foci count in DM1 hiNeurons of one sample. Counting was performed manually. n = 3, a total of 324 cells were analyzed per sample. Paired t-test was used to compare the means of cytoplasmic and nuclear RNA foci count in DM1 hiNeurons. *P< 0.05. (b) Box and whisker plot displays the results of cytoplasmic RNA foci count in placebo and ACT treated DM1 hiNeurons. Each sample is presented in different color. Line and (+) sign inside the box represent median and mean of replicates (outcome analyzed), respectively. Whiskers show minimum & maximum values. (c) Graph shows percentage of cytoplasmic RNA foci reduction in DM1 hiNeurons by ACT treatment. (d) Scatter plot shows percentage of DM1 hiNeurons containing cytoplasmic RNA foci in placebo and ACT treatment groups. Each symbol represents the percentage of DM1 hiNeurons containing cytoplasmic RNA foci per sample replicate. Line represents the mean. Counting was performed manually. n = 3 for each group, a total of 324 nuclei were analyzed per sample. ns, not significant compared by repeated measures one-way ANOVA test. P, placebo (same amount of diluent without drug).
(TIF)

S2 Fig Tolerability of ctrl and DM1 hiNeurons to the studied doses of ACT. (a and c) Live cell images of untreated ctrl and DM1 hiNeurons at 9 DPI, respectively. (b and d) Live cell images of ctrl and DM1 hiNeurons after 24 h treatment with placebo (left), 100 nM ACT (middle) or 200 nM ACT (right). Good tolerability was observed at 100 nM ACT in ctrl and DM1 hiNeurons whereas some cytotoxicity was observed in 200 nM ACT treated cells. Scale bar, 50 μm.
(TIF)

S3 Fig Tolerability of ctrl and DM1 hiNeurons to the studied doses of erythromycin lactobionate. (a and c) Live cell images of untreated ctrl and DM1 hiNeurons at 8 DPI, respectively. (b and d) Live cell images of ctrl and DM1 hiNeurons after 48 h treatment with placebo (left), 35 μM (middle) or 65 μM erythromycin (right). Good tolerability was observed at 35 μM erythromycin in ctrl and DM1 hiNeurons whereas some cytotoxicity was observed in 65 μM erythromycin treated DM1 hiNeurons. Scale bar, 50 μm.
(TIF)
