# Supplementary material for: Pharmacotherapy alleviates pathological changes in human direct reprogrammed neuronal cell model of myotonic dystrophy type 1
Source: PLoS One. 2022 Jul 1;17(7):e0269683. doi: 10.1371/journal.pone.0269683 (PMC9249217; doi:10.1371/journal.pone.0269683)
Supplement: S1 Table — (PDF) [file pone.0269683.s001.pdf]

Supplementary Table 1. List of primers used for RT-PCR:

Genes (exon)	Forward primer Sequence(5' to 3')	Reverse primer Sequence(5' to 3')	Annealing temperature used	PCR cycles	DNA fragment size
*MBNL*1 (exon 5) [1]	AGGGAGATGCTCTCG GGAAAAGTG	GTTGGCTAGAGCCT GTTGGTATTGG	53 ºC	31	–e5 ~225 ± 5%[a]<br>+e5 ~280 ± 5%
*MBNL*2 (exon 5) [1]	ACAAGTGACAACACC GTAACCG	TTTGGTAAAGGATGA AGAGCACC	53 ºC	35	–e5 ~225 ± 5%<br>+e5 ~280 ± 5%
*MBNL*2 (exon 7,8) [2]	CACGCCGCGTTCATT CCAAC	TAGCATGCAGTTTGT GGCAA	61 ºC	31	-e7,-e8 ~155 ± 5%<br>+e7,-e8 ~185 ± 5%<br>-e7,+e8 ~245 ± 5%<br>+e7,+e8 ~285 ± 5%
MAPT (exon 2,3) [3]	TACGGGTTGGGGGA CAGGAAAGAT	GGGGTGTCTCCAAT GCCTGCTTCT	55 ºC	35	-e2,-e3 ~125 ± 5%<br>+e2,-e3 ~210 ± 5%<br>+e2,+e3 ~300 ± 5%
CSNK1D (exon 9) [2]	GATACCTCTCGCATG TCCACCTCACA	GCATTGTCTGCCCTT CACAGCAAT	61 ºC	33	–e9 ~120 ± 5%<br>+e9 ~180 ± 5%
MPRIP (exon 9) [4]	GCACATGGAGACCAA TGCAGTGG	GCTTAGTCAGCCAG CCTTTCTTGA	55 ºC	31	–e9 ~140 ± 5%<br>+e9 ~255 ± 5%

[a]± 5% indicates sizing accuracy of DNA-500 kit used with MultiNA automated microchip electrophoresis system.

References:

1. Jenquin JR, Yang H, Huigens RW 3rd, Nakamori M, Berglund J. Combination Treatment of Erythromycin and Furamidine Provides Additive and Synergistic Rescue of Mis-Splicing in Myotonic Dystrophy Type 1 Models. ACS pharmacology & translational science. 2019;2(4):247–263. doi:10.1021/acsptsci.9b00020
2. Nishi M, Kimura T, Igeta M, Furuta M, Suenaga K, Matsumura T et al. Differences in splicing defects between the grey and white matter in myotonic dystrophy type 1 patients. PloS one. 2020;15(5):e0224912. doi:10.1371/journal.pone.0224912
3. Leroy O, Wang J, Maurage CA, Parent M, Cooper T, Buée L et al. Brain-specific change in alternative splicing of Tau exon 6 in myotonic dystrophy type 1. Biochimica et biophysica acta. 2006;1762(4):460–467. doi:10.1016/j.bbadis.2005.12.003
4. Suenaga K, Lee K, Nakamori M, Tatsumi Y, Takahashi M, Fujimura H et al. Muscleblind-like 1 knockout mice reveal novel splicing defects in the myotonic dystrophy brain. PloS one. 2012;7(3):e33218. doi:10.1371/journal.pone.0033218
