# Supplementary figures and images for: Pharmacotherapy alleviates pathological changes in human direct reprogrammed neuronal cell model of myotonic dystrophy type 1
Source: PLoS One. 2022 Jul 1;17(7):e0269683. doi: 10.1371/journal.pone.0269683 (PMC9249217; doi:10.1371/journal.pone.0269683)

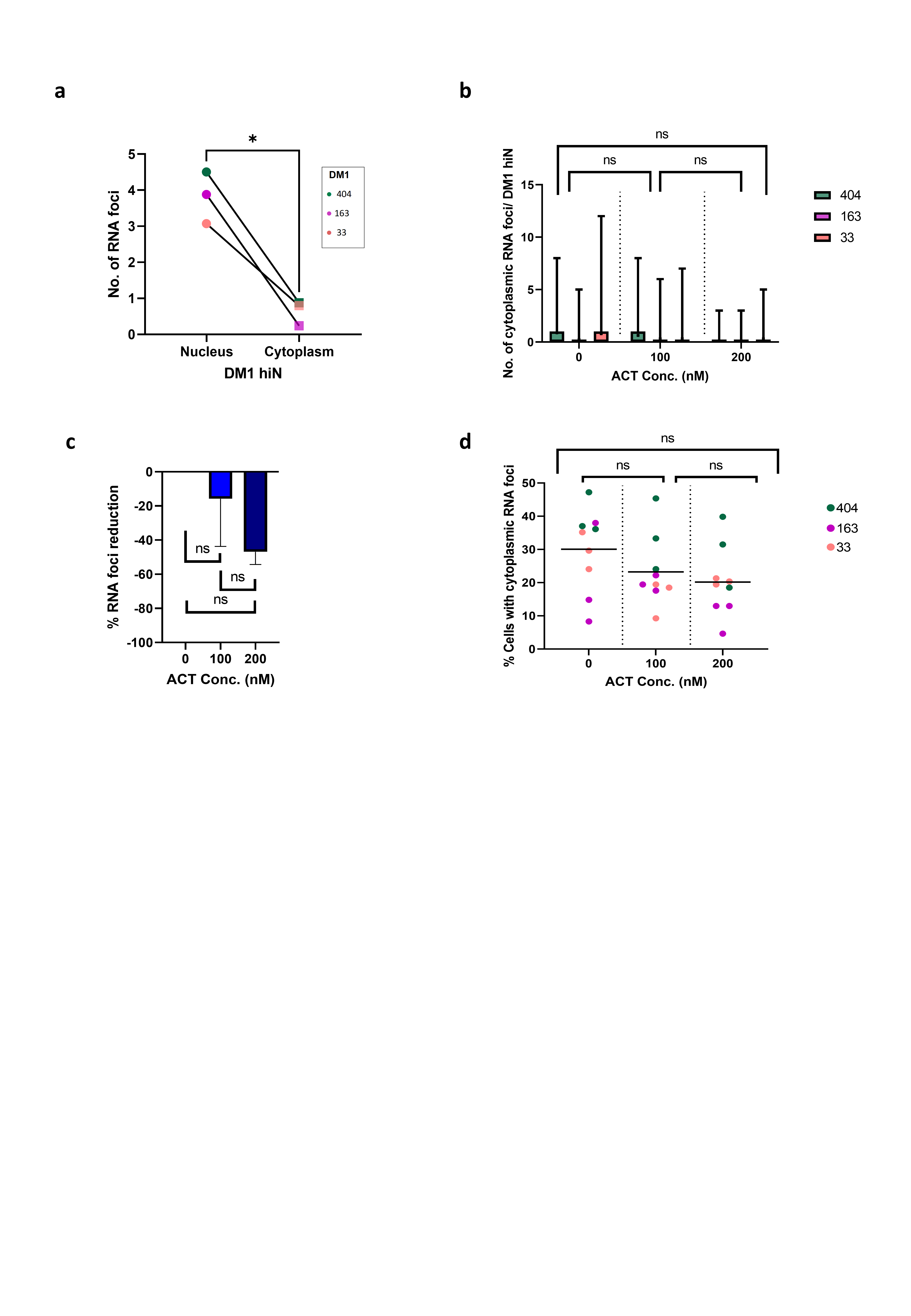

Supplement: S1 Fig — (a) Scatter plot shows number of RNA foci per nucleus vs cytoplasm of DM1 hiNeurons. Each symbol represents the mean of RNA foci count in DM1 hiNeurons of one sample. Counting was performed manually. n = 3, a total of 324 cells were analyzed per sample. Paired t-test was used to compare the means of cytoplasmic and nuclear RNA foci count in DM1 hiNeurons. *P< 0.05. (b) Box and whisker plot displays the results of cytoplasmic RNA foci count in placebo and ACT treated DM1 hiNeurons. Each sample is presented in different color. Line and (+) sign inside the box represent median and mean of replicates (outcome analyzed), respectively. Whiskers show minimum & maximum values. (c) Graph shows percentage of cytoplasmic RNA foci reduction in DM1 hiNeurons by ACT treatment. (d) Scatter plot shows percentage of DM1 hiNeurons containing cytoplasmic RNA foci in placebo and ACT treatment groups. Each symbol represents the percentage of DM1 hiNeurons containing cytoplasmic RNA foci per sample replicate. Line represents the mean. Counting was performed manually. n = 3 for each group, a total of 324 nuclei were analyzed per sample. ns, not significant compared by repeated measures one-way ANOVA test. P, placebo (same amount of diluent without drug). (TIF) [file pone.0269683.s002.TIF]

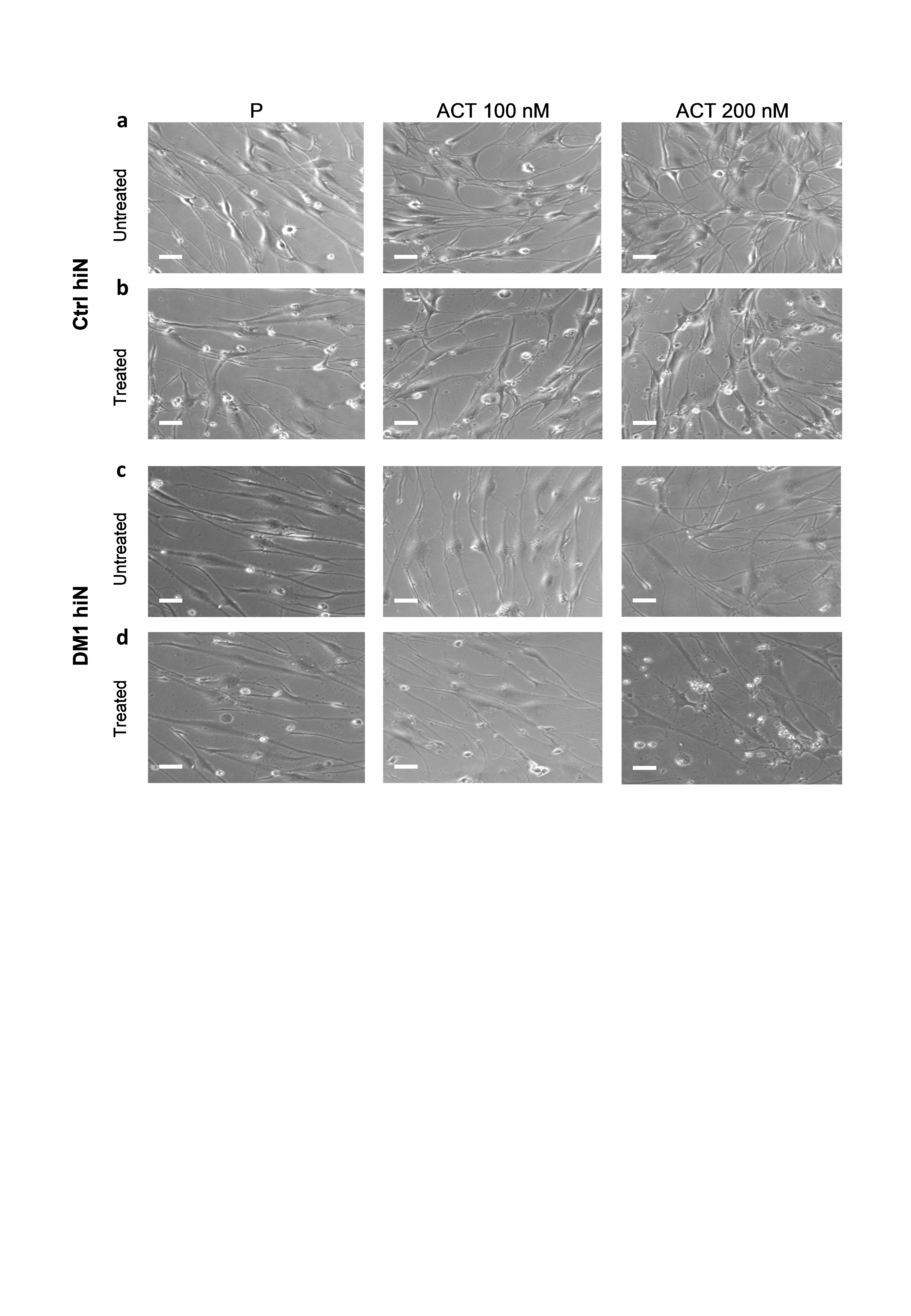

Supplement: S2 Fig — (a and c) Live cell images of untreated ctrl and DM1 hiNeurons at 9 DPI, respectively. (b and d) Live cell images of ctrl and DM1 hiNeurons after 24 h treatment with placebo (left), 100 nM ACT (middle) or 200 nM ACT (right). Good tolerability was observed at 100 nM ACT in ctrl and DM1 hiNeurons whereas some cytotoxicity was observed in 200 nM ACT treated cells. Scale bar, 50 μm. (TIF) [file pone.0269683.s003.TIF]

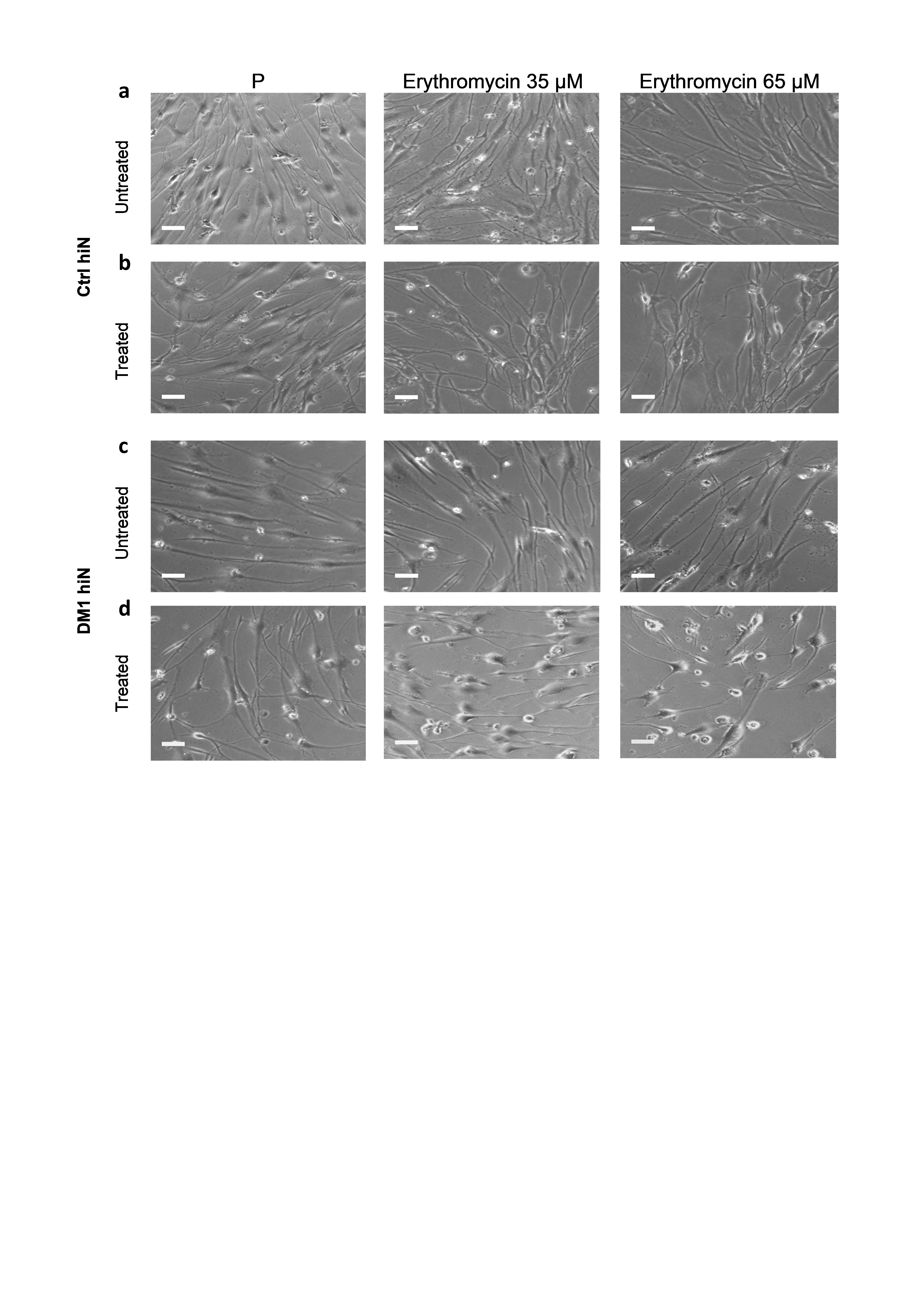

Supplement: S3 Fig — (a and c) Live cell images of untreated ctrl and DM1 hiNeurons at 8 DPI, respectively. (b and d) Live cell images of ctrl and DM1 hiNeurons after 48 h treatment with placebo (left), 35 μM (middle) or 65 μM erythromycin (right). Good tolerability was observed at 35 μM erythromycin in ctrl and DM1 hiNeurons whereas some cytotoxicity was observed in 65 μM erythromycin treated DM1 hiNeurons. Scale bar, 50 μm. (TIF) [file pone.0269683.s004.TIF]
